# Factors Influencing the Initiation and Continued Engagement of Digital Mental Health Tools Among Adults: Theory of Planned Behavior–Informed Systematic Review

**DOI:** 10.2196/88731

**Published:** 2026-05-15

**Authors:** Nan Cheng, Mary K Lam, Christine Grove, Monica Wachowicz

**Affiliations:** 1 School of Health and Biomedical Sciences, STEM College, Royal Melbourne Institute of Technology (RMIT) University, 225-245 Plenty Road, Bundoora, Victoria, 3082, Australia, +61 3 9925 2000; 2 School of Science, STEM College, Royal Melbourne Institute of Technology (RMIT) University, Bundoora, Victoria, Australia

**Keywords:** digital mental health, engagement, Theory of Planned Behavior, usability, access, stigma, equity, qualitative synthesis

## Abstract

**Background:**

Digital mental health tools (DMHTs) offer scalable support, but engagement varies. Understanding the shapes of initiation and ongoing use is essential for effective design and implementation.

**Objective:**

This study aims to synthesize determinants of adults’ initiation and engagement with DMHTs, organized through two lenses: (1) psychological factors aligned with the theory of planned behavior (TPB) and (2) design and access features.

**Methods:**

A systematic search of 9 databases (June 2025) identified qualitative and mixed methods primary studies reporting end-users’ experiences with DMHTs. Studies were screened and reported in accordance with the PRISMA (Preferred Reporting Items for Systematic Reviews and Meta-Analyses) guidelines. Quality appraisal used quality assessment with diverse studies (QuADS). Data were synthesized using a framework-guided thematic approach, mapping findings to TPB constructs and complementary design and access domains.

**Results:**

A total of 22 studies met inclusion criteria. Findings clustered into 2 interdependent domains. TPB constructs explained how beliefs, social expectations, and perceived control shaped decisions to start and persist with DMHTs. Design and access features frequently acted through these same pathways, especially by altering perceived behavioral control (PBC), with cost, connectivity, device constraints, and time flexibility affecting feasibility, with content design and privacy shaping perceived value and trust. Perceived fit (goals, cultural or linguistic relevance, and routine alignment) consistently influenced both initiation and continuation. Several features operated bidirectionally; depending on context, the same feature could facilitate or hinder engagement.

**Conclusions:**

Engagement with DMHTs is jointly determined by users’ beliefs and the design and access conditions within which tools are offered. Implementation should pursue a dual strategy, strengthening willingness to seek support (addressing attitudes, norms, and perceived control) while engineering low-effort, trustworthy, and context-appropriate experiences. Priorities include equity-focused policies (data costs, devices, and connectivity), transparent data practices, co-design with diverse communities, and consistent, theory-informed outcome measures.

## Introduction

### Background

Common psychological distress and mental health concerns are widespread across populations, affecting large numbers of people at different life stages and placing a sustained burden on well-being and daily functioning [1]. Despite the high lifetime prevalence, a substantial treatment gap persists in traditional in-person services. Multiple, interacting constraints shape whether people initiate help-seeking. At the social level, stigma and limited visible role models for successful help-seeking can suppress motivation to approach services [2]. This absence of relatable exemplars is often more pronounced in cultural or contextual minority communities where norms do not encourage formal mental health care, which can reduce perceived usefulness or necessity and weaken attitudes toward help-seeking, with stigma and the fear of loss of face further suppressing intentions to seek support [3,4]. Regarding the psychological domain, beliefs about effectiveness and low confidence in navigating and accessing the most appropriate care can inhibit first contact [5,6]. Structurally, limited-service availability, travel time, out-of-pocket costs, and workforce shortages reduce practical access, especially in rural and resource-constrained settings [7]. Together, these barriers contribute to delayed or foregone care, meaning that people who could benefit do not receive support or receive it late.

Technology, as a rapidly expanding field, has been recognized as a promising approach for enhancing access to mental health care. Digital mental health tools (DMHTs) are proposed as one such approach, offering flexible, private, and lower-cost entry points that can complement in-person services [8].

### DMHTs

DMHTs refer to technology-enabled support for mental health information, assessment, and self-management delivered through channels such as mobile apps, web platforms, SMS text messaging services, and chatbots [9,10]. With continuing advances in digital technology, the use of these tools within health care has expanded rapidly across countries and service contexts, spanning psychoeducation, symptom monitoring, structured therapeutic modules, peer support, crisis response, and tele-mental health [11,12]. In this review, DMHTs are considered in the context of adult users seeking self-care and well-being support rather than clinical treatment.

During the COVID-19 pandemic, research observed significant increases in the uptake of digital services, underscoring their potential role in maintaining access to support when traditional pathways are constrained [13]. In that review, self-guided courses showed the highest uptake across offerings, indicating demand for direct-to-public, on-demand options. Prior research has also highlighted benefits of self-guided DMHTs, such as anonymity, convenience, affordability, and scalability, which may be especially important for first steps toward support [14].

Across populations, DMHTs are frequently described as lowering perceptual and practical barriers to early support by offering more discreet, flexible, and place-independent access to help [12,15,16]. This potential appears especially valuable where stigma and cultural pressure inhibit help-seeking. Gendered expectations illustrate this dynamic. Studies consistently report that men face stronger stigmatizing norms around seeking professional help, rooted in beliefs that managing distress independently signals strength while seeking support signals weakness [17,18]. DMHTs may soften these pressures by allowing users to engage privately and at times and locations that feel safer than in-person services, including use at home, thereby preserving a sense of anonymity and control [19,20].

In communities where collective reputation and deference to public opinion are highly valued, fears of discrimination, “losing face,” and public stigma can deter formal help-seeking [12,21]. In such settings, accessible psychoeducation delivered digitally can improve mental health literacy, clarify symptoms and treatment options, and challenge stigmatizing beliefs, which in turn can strengthen intentions to seek help [22,23].

However, the potential benefit of DMHTs can only be achieved when individuals are willing and able to initiate and sustain engagement [24]. While DMHTs offer clear advantages in accessibility and flexibility, public acceptability remains mixed. Preexisting doubts about the effectiveness or rigor of digital interventions, along with prior negative experiences, are linked to lower motivation to try or persist with DMHTs [25,26]. When users view digital delivery as inferior to face-to-face care, they tend to rate tools as lower in quality, effectiveness, and efficiency, which undermines uptake. Persistent digital exclusion among minority and marginalized communities has also been documented [27]. A lack of culturally responsive content and insufficient attention to the needs of specific ethnic communities can further undermine perceived relevance and increase the burden of engagement [28]. Privacy and confidentiality concerns, especially uncertainty about data storage and sharing practices, are also prominent barriers [12,15]. Although privacy worries are not unique to digital contexts, remote services introduce distinct data risks that many users perceive as heightened and difficult to evaluate. Confidence in navigating digital tools further impacts individuals’ willingness to initiate use and to sustain engagement. When users have limited prior experience with self-initiated help or receive minimal guidance, complex or cluttered interfaces can quickly overwhelm them, lowering their willingness to continue [15,29].

Existing reviews on the use of DMHTs have tended to focus on tool formats and design characteristics [30-32] or on outcomes such as clinical efficacy and cost-effectiveness [33,34]. Syntheses that center specifically on determinants of user initiation and early engagement remain limited. Where engagement has been discussed, psychological determinants are rarely organized within a coherent theoretical framework [12,15,35]. Moreover, although theory has long been used to explain general help-seeking behavior, theory-informed examinations focused explicitly on digital mental health services are comparatively rare [36,37].

This study focuses on initiation and early engagement and integrates a theory-driven lens by applying the theory of planned behavior (TPB) to synthesize how attitudes, subjective norms, and perceived behavioral control (PBC) operate as barriers or facilitators in DMHT contexts. The review also analyzes design and access features in parallel and explores how these features intersect with perceived control. To our knowledge, this is the first systematic review to jointly examine theoretical and practical determinants of adults’ help-seeking intentions and early engagement with DMHTs.

### The TPB

We position the TPB as the primary lens for interpreting initiation and early engagement with DMHTs, while acknowledging the relevance of other behavior-change frameworks. In TPB, behavior is shaped by 3 proximal determinants, including attitudes toward the behavior, perceived social expectations (subjective norms), and the sense that one can carry out the behavior given available skills and opportunities (PBC) [38,39]. These determinants influence intentions, and intentions in turn guide behavior [40].

TPB has been widely used to predict health-related behaviors, and, in the mental health field, it has most commonly been applied to understand the factors shaping help-seeking intentions and behaviors [41]. In particular, it has informed the early stages of mental health support by clarifying how willingness to seek professional help may be influenced. Early work also applied TPB to examine doctors’ intentions to provide support within mental health services, highlighting its value for understanding clinical decision-making and its potential to improve service provision [42]. To date, TPB has been widely used in mental health research to examine help-seeking across a range of mental health concerns. Across settings, studies have consistently identified positive associations between TPB constructs, particularly attitudes, subjective norms, and perceived behavioral control, and intentions to seek professional psychological support [43,44]. In digital mental health, although relatively few DMHTs have been explicitly developed on the basis of TPB, the framework has increasingly been used to inform the evaluation of DMHTs and interventions. In this context, TPB has most often been applied to better understand, predict, and support users’ initial uptake and sustained engagement with digital interventions [45,46].

Other frameworks of behavior theories have also been used in previous studies to assist mental health services researchers. These include the Health Belief Model, which centers on individual beliefs and attitudes, perceived benefits and barriers, and cues to action and is frequently used in adherence research, including medication adherence [47]. Moreover, Social Cognitive Theory, which emphasizes outcome expectations, observational learning, and self-efficacy, is commonly used to inform skills training or disease-management interventions for serious mental illness [48].

TPB is adapted in this study, as it is aligned with this review’s scope. This study focuses on adults without a prior diagnosis using DMHTs for general well-being and self-care. Early decisions to try a tool and to continue beyond initial contact are intention-sensitive moments, for which TPB provides a concise account. Moreover, TPB maps onto key DMHT concerns; attitudes capture evaluations of content credibility, privacy, and perceived helpfulness; subjective norms reflect the influence of peers, family, and clinicians as well as broader stigma; and PBC links directly to usability, cost, connectivity, and time flexibility that condition whether people feel able to engage. We read study findings through the 3 TPB constructs to clarify how participants and the original authors understand the role of beliefs, social influence, and control in shaping intentions to initiate and sustain early engagement with DMHTs.

The limitations of TPB in digital mental health contexts are also recognized. Although TPB offers a useful framework for understanding intention-related processes, it gives less prominence to affective and automatic influences (for example, mood and habit), the intention-to-behavior gap, and broader structural or logistical determinants that may shape access to care [40]. TPB is therefore used in this review as a flexible interpretive guide rather than a strict predictive model. Within this framing, design and access-related determinants, although not directly specified within TPB, would be considered in terms of how they may interact with TPB-informed factors by shaping users’ perceptions, evaluations, and sense of control and influence initiation and engagement with DMHTs.

### This Study

This review synthesizes factors influencing user initiation and engagement with DMHTs through 2 connected lenses, including psychological factors directly aligned with TPB and practical design and access features that may influence or intersect with TPB-informed factors. The review is guided by two questions:

- Review question 1: within the TPB, how do attitudes, subjective norms, and PBC function as barriers or facilitators of DMHT use?
- Review question 2: across design and access domains, what factors function as barriers or facilitators of DMHT use?

The scope of this study centers on adults who engage with DMHTs for self-care and well-being rather than clinical treatment. We consider tools intended for general mental health support rather than those targeted to a specific diagnosed condition. Consistent with this focus, we include primary studies that examine users’ experiences, intentions, and engagement with tools and exclude papers whose primary aim is to test treatment efficacy for diagnosed disorders or to evaluate clinical outcomes.

Although prior reviews have examined determinants of engagement with DMHTs [15,49], and TPB has been reviewed in relation to mental health help-seeking [41,50], to the best of our knowledge, none of the reviews have integrated a TPB-aligned synthesis of psychological determinants alongside a parallel analysis of design and access features specific to initiation and engagement with DMHTs. This dual-lens approach allows us to distinguish attitudinal and normative targets for communication from engineering, service, and policy levers that shape real-world opportunity and effort.

By mapping evidence to TPB constructs and, in parallel, to concrete product and context features, this review aims to present findings on factors that shape first use and continuation. This will inform future development of DMHTs by translating qualitative insights into actionable priorities for product design and delivery, including usability, privacy assurances, and barriers related to cost or connectivity. For policymakers, this study outlines implications for raising awareness, reducing stigma, and ensuring the resources and infrastructure needed to facilitate equitable access and engagement.

## Methods

### Overview

This review was designed and reported in accordance with established methodological guidance for evidence syntheses of qualitative and mixed methods research, incorporating Population, Intervention, Comparator, and Outcome (PICO) for search formulation, PRISMA (Preferred Reporting Items for Systematic Reviews and Meta-Analyses; Checklist 1) for study screening and reporting, and quality assessment with diverse studies (QuADS) for quality appraisal [51-53]. The review focused specifically on primary research reporting end users’ experiences with DMH services and tools, enabling the synthesis of barriers and facilitators to initiation and ongoing use.

### Search Strategy

In June 2025, 9 databases were systematically searched, including PubMed/MEDLINE, Embase, PsycINFO, Scopus, Cochrane Central Register of Controlled Trials, CINAHL Complete, Web of Science Core Collection, ACM Digital Library, and Google Scholar. Search strategies combined database subject vocabulary with free text terms for digital mental health services and tools, user experience and engagement, barriers and facilitators, and adult populations. Before screening commenced, a research librarian reviewed the database coverage and draft strategy. The consultation followed the Peer Review of Electronic Search Strategies (PRESS) guideline to enhance sensitivity and precision; minor refinements were made to improve the strategy [54]. Search strings were drafted in PubMed/MEDLINE and translated to the syntax of each database; full database-specific strings are provided in Multimedia Appendix 1. Gray literature searching followed established recommendations; the first 200 relevance-ranked Google Scholar results were screened for eligibility [55].

The PICO framework was used as the conceptual starting point for formulating the review question in a health context and drew on 2 extensions that better matched this topic: Population, Intervention, Comparator, Outcome, and Context (PICOC) to make the contextual boundary explicit and Population, Intervention, Comparator, Outcome, and Study design (PICOS) to prespecify eligible study designs [51,56,57]. Operational definitions were as follows. The population of interest was adults without a recorded diagnosis of a mental health condition at the time of participation. The intervention of interest was digital mental health services and tools, including websites and mobile apps that deliver psychoeducation, self-help content, or other user-directed support. Outcomes were users’ experiences of digital services and tools, including perceived barriers and facilitators, acceptability, intention to use, engagement, and adherence. The context was early-stage help seeking and use outside clinical service delivery; services or tools designed for clinical service delivery were excluded, as clinical use of digital mental health support is outside this review’s scope. Eligible study designs were those that collected primary data directly from end users. Secondary analyses, review studies, protocols, editorials, and narrative opinion pieces were not eligible. Although review articles were excluded from the synthesis, their reference lists were screened to identify additional eligible primary studies through backward citation chasing.

### Study Selection

Records identified through the database searches were imported into Covidence, a web-based collaboration platform for deduplication and screening. Study selection followed the PRISMA recommendations, with a PRISMA flow diagram presenting the document identification, screening, eligibility assessment, and inclusion [52]. No limits were applied regarding geography, race, gender, culture, or publication year at selection; the only limit applied during the search was the English language.

Screening proceeded in 2 stages against the prespecified eligibility criteria (Table 1). At the title and abstract stage, the first authors (NC) screened records to remove clearly ineligible studies. Articles judged potentially relevant were retrieved in full. At the full text stage, the first and second authors (NC and MKL) independently assessed each article for final eligibility. Studies were excluded at full text if they included ineligible populations such as participants younger than 18 years or clinical cohorts with diagnosed mental health conditions (marked as “Ineligible participant population” in the PRISMA diagram); if outcomes did not report users’ initial access to digital mental health care or users’ experiences while interacting with the digital service or tool (“Irrelevant outcomes”); if the intervention was not a digital mental health service or tool, including studies limited to traditional in-person services or general mental health services without specific findings on digital initiation or use (“Ineligible intervention”); or if the context was clinical service delivery, including digital tools used within formal treatment or case management (“Ineligible context”). Any disagreements between reviewers were resolved through discussion, and when consensus could not be achieved, a third reviewer (CG) aided in the resolution.

**Table 1.** Characteristics of the included studies (N=22).

Characteristics	Values^a^, n (%)
Country	
United States	6 (27)
United Kingdom	3 (14)
Australia	3 (14)
Ireland	2 (9)
Sweden	2 (9)
Other countries (Canada, Hong Kong [China], Philippines, Korea, Singapore, Egypt, and Uganda)	7 (32)
Study design	
Semistructured interviews	10 (45)
Survey	8 (36)
Interview+ survey	3 (14)
Group discussion	1 (5)
DMHT^b^ type	
Web-based program	9 (41)
Mobile app	6 (27)
Telehealth	3 (14)
AI chatbot	1 (5)
Target mental health concern	
General well-being	17 (77)
Depression	5 (23)
Anxiety	3 (14)
Distress	1 (5)
Self-harm	1 (5)

a Some studies reported more than one type of digital tool, mental health concern, or participant demographic category, while others did not report specific information. As such, the total number of mentions and percentages may exceed or fall short of 100%.

b DMHT: digital mental health tool.

### Quality Assessment

Methodological quality was appraised using the QuADS instrument, which is suitable for reviews synthesizing qualitative, quantitative, and mixed methods evidence [53]. This tool has been used in previous systematic review studies that assessed qualitative data in the field of mobile health tools [58]. QuADS comprises 13 criteria scored on a 4-point scale from 0 (not reported) to 3 (explicit and detailed), yielding a total score between 0 and 39. For each included study, we calculated a percentage score by dividing the obtained total by the maximum possible score. Consistent with prior reviews, we classified studies as poor (<25%), moderate (25%‐49.9%), good (50%‐74.9%), or high quality (≥75%). Item-level QuADS scores and study-level classifications are provided in Multimedia Appendix 2.

### Data Extraction and Synthesis

Structured extraction spreadsheets were used to capture study characteristics (design, setting, and country), participant characteristics, details of the digital mental health tool, and all author interpretations and participant quotations relevant to initiation, use, engagement, and continuation of tool use. Extraction was conducted in NVivo (version 15; Lumivero) to support coding and traceability of evidence to source texts, with a companion spreadsheet to organize study identifiers, raw excerpts, code labels, and evolving theme definitions (Multimedia Appendix 3).

An abductive thematic analysis was undertaken. The initial coding framework was deductively informed by the TPB to organize evidence under attitude, subjective norms, and PBC [59]. During the review, practical constraints such as the technical and logistical access–related factors emerged frequently and suggested shaping real-world use of digital tools [60,61]. With the abductive approach, data aligned with TPB were collected and sensitively analyzed while remaining open to patterns that fell outside the TPB framework. This approach is appropriate for this study because our aim was 2-fold: to examine internal factors that inform an individual’s intention to seek help through digital tools (for which TPB provides a well-established lens) and to identify practical conditions that shape tool uptake and continued use in everyday settings, which are not fully encompassed by TPB alone.

The first author (NC) coded all included evidence and wrote analytic memos throughout. The coding and theme development followed the 6 phases described by Braun and Clarke [62], including familiarization, initial coding, searching for themes, reviewing themes, defining and naming themes, and producing the synthesis. To enhance validity and reliability within a single-coder workflow, which is common and defensible in qualitative evidence syntheses, we used safeguards, including maintaining an explicit codebook with definitions and inclusion and exclusion rules, linking every code to verbatim evidence to ensure auditability, and holding regular consensus meetings, in which the second and third authors (ML and CG) reviewed exemplar extracts for each code and theme and provided feedback, leading to clarification and refinement of the coding frame [15,63,64].

For classification of determinants, we defined a factor as a barrier or a facilitator when an included paper explicitly labeled it as such or when author interpretations or participant quotations clearly described the factor as hindering or enabling initiation, use, engagement, or continuation. For example, “Another hindrance to access to DMH interventions cited is the high cost of DMH apps” [60] and “Participants also stated that it was too expensive as follows: I’m not sure I would pay $10 a month for it. when life was crazy” [65]. In both instances, costs are identified as barriers.

To aid transparency, we report simple counts of how often a code or theme appeared across the included studies. These counts describe reporting prevalence rather than importance, magnitude, or causal influence. Consistent with guidance on qualitative synthesis, salience was judged on the quality and richness of supporting evidence, the coherence of the pattern across contexts, and the contribution of a theme to explaining variation, not on frequency alone [62,66].

## Results

### Search and Screening Results

A total of 1668 records were identified through comprehensive searches across 9 databases, including PubMed (n=664), Embase (n=153), PsycINFO (n=168), Scopus (n=216), Cochrane Central Register of Controlled Trials (n=16), CINAHL Complete (n=8), Web of Science (n=112), ACM Digital Library (n=181), and Google Scholar (n=150; see Table 2). The search strategy incorporated key terms related to DMHTs, early-stage mental health support, and adult populations. Boolean operators and truncation were used to optimize retrieval, with minor adaptations made to fit the indexing structures of individual databases.

**Table 2.** Inclusion and exclusion criteria.

Criterion	Inclusion criteria	Exclusion criteria
Population	Adults aged ≥18 years; general population, not currently engaged in formal/professional mental health treatment	Individuals <18 years; clinical populations already receiving professional care (eg, ongoing therapy, inpatient treatment); participants with diagnosed psychiatric disorders under active treatment; studies reporting exclusively professional/clinician perspectives without user-level data
Intervention	Digital mental health services/tools designed to support engagement, including: Mental health chatbots Mobile mental health applications Web-based programs Guided self-help tools Tele-mental health platforms	Studies focusing on general digital health without specific evaluation of digital mental health tools; nondigital or face-to-face-only interventions; tools designed exclusively for long-term treatment or relapse prevention, beyond the scope of help-seeking and engagement
Context	Mental health support in the initial or ongoing engagement stages, such as: Psychoeducation Symptom recognition Tools facilitating help-seeking motivation or service initiation	Studies centered on long-term treatment adherence, clinical outcomes, or relapse prevention as primary outcomes; school-based or workplace programs that do not assess individual-level help-seeking or engagement
Outcomes	Barriers and/or facilitators of digital mental health service use User experience and engagement (initial and continuance) Trust, stigma, and perceptions Cultural acceptability and relevance Attitudes toward digital mental health tools	Studies not addressing barriers/facilitators or relevant user perspectives; outcomes limited to clinical efficacy without consideration of access, acceptability, or engagement; studies presenting perspectives on general (nondigital) mental health services only
Study design	Primary research presenting user-level data, including: Qualitative (eg, interviews and focus groups) Mixed methods studies Observational studies Survey-based studies	Systematic reviews, meta-analyses, editorials, or theoretical/commentary papers; studies reporting only provider/clinician perspectives without user voice

After removing 362 duplicates (357 through Covidence [Veritas Health Innovation] and 5 manually), 1306 records remained for title and abstract screening. Of these, 1212 records were excluded for not meeting the eligibility criteria, primarily due to lack of relevance to digital tools targeting adults in early-stage mental health support.

The full texts of 94 studies were assessed, resulting in the exclusion of 72 studies. Reasons included participants aged 18 years and younger (n=10), absence of relevant outcomes on factors influencing initial access to DMHTs (n=6), use of ineligible interventions such as traditional or in-person mental health services (n=23), general digital health services without a mental health focus (n=19), studies centered on clinical interventions (n=12), unavailable full-text (n=1), and ineligible publication types such as theses (n=1).

**Figure 1.**
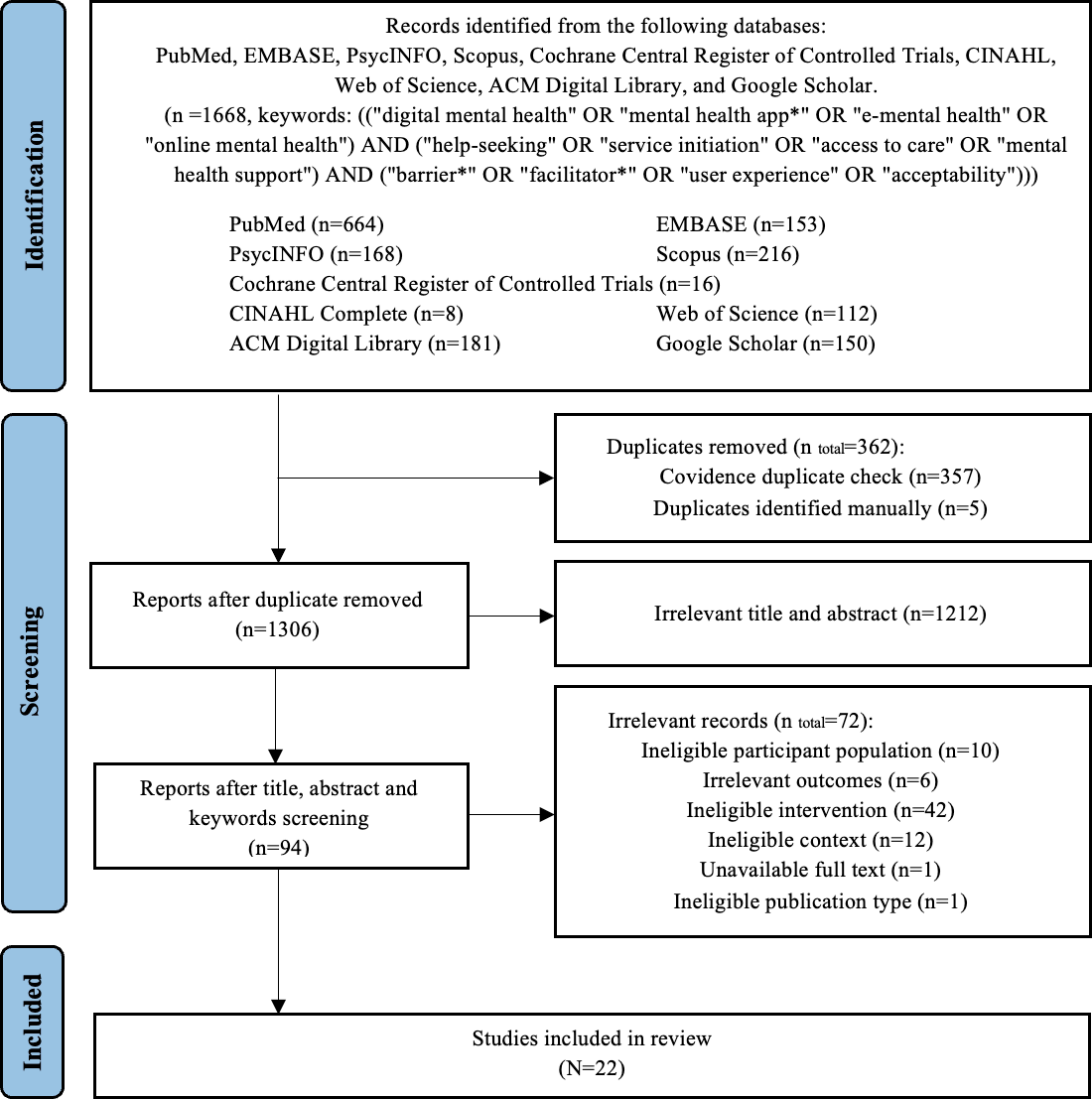
PRISMA (Preferred Reporting Items for Systematic Review and Meta-Analyses) flow diagram.

Ultimately, 22 [28,60,61,65,67-84] studies met the inclusion criteria and were included in the final synthesis (Figure 1).

### Overview of the Included Articles

The 22 included studies were conducted across 12 countries, reflecting a broad range of cultural and ethnic contexts, with the majority based in the United States. Sample sizes ranged from 10 to 5556 participants, with ages spanning from 18 to 107 years. Most studies used a qualitative design, primarily using semistructured interviews to collect participants’ perspectives. The DMHTs examined included web-based programs, mobile apps, telehealth services, and chatbots. The most commonly targeted concerns were general well-being, followed by depression and anxiety.

Detailed information on each reviewed study is presented in Multimedia Appendix 4, and a summary of study characteristics and key findings is provided in Table 3.

**Table 3.** Themes aligned with theory of planned behavior constructs.

Theme and subtheme	Example Quote	Value^a^, n (%)
Attitude and individual’s evaluations		9 (41)
Negative attitudes toward mental health help-seeking	“People perceive seeking help for a mental health problem as a sign of weakness rather than a strength” [60] “When presented with the idea of professional help, they immediately say no or something like ‘why do you think I’m like that?’” [69]	4 (18)
Mistrust toward digital tools	“Many participants had low expectations for how useful the intervention was going to be.” [74] “strong negative attitudes toward apps (ie, apps aren’t helpful, are harmful)” [77]	3 (14)
Belief-based rejection	“Many participants believed that depression cannot be prevented” [70] “Other strategies work better (for example: praying, alcohol)” [28]	3 (14)
Subjective norms		17 (77)
Stigma	“Many participants in the study revealed that they felt ashamed of their mental distress and feared the judgment of others, feelings which had previously prevented them from seeking help or even talking about their mental health” [74] “negative perceptions, myths, and misconceptions associated with mental illness” [60]	16 (73)
Cultural belief	“Sadness and other dysphoric emotions were broadly classified as unhealthy and unwanted” [74] “We have been brought up as Africans, we believe these things [mental health] don’t exist” [60]	3 (14)
Perceived social support	“Conversations with family, in particular a partner or spouse, were seen as the impetus for accessing the DMHS^b^ for the initial online assessment” [71] “Hearing from peers with experience of perceived mental health support allowed for emotional release” [69]	3 (14)
Perceived behavioral control		14 (64)
Autonomy and Self-determination	“Just turn your computer on and do it that way, it’s not quite as difficult to get yourself to do it” [74] “My mental state has deteriorated. I just have no determination or willpower to do anything” [70]	5 (23)
Awareness	“Over half of participants did not know that mental health websites and apps existed” [78] “Many young [people] don’t know that these platforms are there. So, [when] they get problems, they don’t know where to run to” [60]	7 (32)
Perceived fit	“Participants shared they would only seek help if the service felt ’relevant’ and ‘practical’” [67] “lack of culturally responsive treatment options and lack of diverse representation within DMHI^c^ surface content” [28]	8 (36)
Knowledge	“Being unsure what to search and look for online instead of searching for hours for a website that I am comfortable with” [81] “Egyptian students were unfamiliar with e-mental health functionalities and technical approaches” [78]	7 (32)

a Value reflects only the number of studies in which each theme was identified and does not imply the relative importance or impact of the themes.

b DMHS: digital mental health service.

c DMHI: digital mental health intervention.

### Quality Assessment

The quality and risk of bias of all included studies were appraised using the QuADS tool (see Multimedia Appendix 2). The methodological quality of the studies ranged from 54% to 87%. Of the total sample, 12 [60,68-71,73,74,76,79-81,84] studies were rated as high quality (77%‐87%), and 10 [28,61,65,67,72,75,77,78,82,84] studies were rated as good quality (54%‐74%). No study was rated as moderate or poor (ie, below 50%).

### Factors Influencing Individual Initiation of DMHTs

To address the review aim of exploring factors influencing individuals’ initiation of DMHTs, a thematic analysis was conducted, guided by the TPB and supported by inductive insights. This abductive analysis resulted in 5 major themes, including attitudes and individual’s evaluation, subjective norms and social expectation, perceived behavioral control, content design and framing, and technical and logistical access.

Each of the 5 themes is introduced with a brief conceptual definition, followed by the corresponding codes and illustrative excerpts, along with the number of studies in which each code was reported. The first 3 themes align directly with TPB constructs, capturing individuals’ evaluations of DMHTs, the influence of social expectations, and perceptions of control over access and usage (see Table 4). The latter 2 themes emerged inductively, highlighting practical and contextual factors shaping users’ experiences with DMHTs, influencing both initiation and sustained engagement.

**Table 4.** Content design and framing factors affecting initiation of digital mental health tools (DMHTs).

Theme and subtheme	Example quote	Value, n (%)
Content design and framing		15 (68)
Content delivery format	“Videos were viewed favorably; students found them easier to engage with than text-based content.” [73] "When asked about how participants wanted the information on the EMH^a^ platform to be delivered, most participants suggested videos explaining mental health topics.” [78]	5 (23)
Linguistic style	“Programs were too text heavy, which deterred participation and caused users to lose interest.” [70] “Simplicity of the intervention as a strength, with one participant stating it was ‘straightforward, and it was just easy to listen to. It wasn‘t like a pain.’” [73]	7 (32)
Embedded features	“Completing an online assessment of their depression and anxiety aided next steps in their mental health treatment journey.” [71] “Self-monitoring quizzes and tailored feedback helped users understand how they were feeling and what could help.” [73]	6 (27)
Human contact options	“The importance of feeling heard and understood by another human was reported widely across the data.” [74] "The value of interacting with others experiencing similar issues was noted, and awareness of the shared experience of others was suggested as a facilitator of further help-seeking.” [75]	11 (27)

a EMH: electronic mental health.

### Attitude and an Individual’s Evaluations

Attitudinal and individual evaluations influence on initiation were identified in 9 [28,60,68-72,74,77] of the 22 included studies. Three key patterns emerged, including negative attitudes toward general mental health, skepticism toward digital health tools, and belief-based rejection of mental health support.

Four [28,60,69,72] studies reported that participants expressed negative attitudes toward seeking professional mental health support more broadly. These attitudes often stemmed from framing help-seeking as a sign of personal weakness. For example, one study noted, *“*People perceive seeking help for a mental health problem as a sign of weakness rather than a strength*”* [60]. Another participant shared, “When presented with the idea of professional help, they immediately say no or something like ‘why do you think I’m like that?’” [69].

In 3 [28,70,71] studies, participants demonstrated predetermined negative attitudes specifically toward DMHTs. These views were often rooted in low expectations of usefulness toward digital tools for managing mental health. One study found, *“*Many participants had low expectations for how useful the intervention was going to be” [74], while another described *“*strong negative attitudes toward apps (ie, apps aren’t helpful, are harmful)” [77].

Moreover, 3 [68,74,77] highlighted how deeply held personal beliefs shaped negative attitudes toward professional mental health support. Some participants doubted the legitimacy or effectiveness of preventive approaches, believing that mental health conditions like depression could not be prevented or treated, or seeking alternative coping strategies. As noted in one study, *“*Many participants believed that depression cannot be prevented*”* [70], and others expressed a preference for alternatives such as “praying” or “alcohol” [28].

### Summary of Individual Evaluation Factors

Negative attitudes toward professional mental health support, often rooted in perceptions of weakness, functioned as barriers. Similarly, skepticism about the usefulness of digital tools limited engagement, with low expectations of effectiveness reinforcing avoidance. In addition, belief-based rejection, such as the view that depression cannot be prevented or the preference for alternative coping strategies, further restricted willingness to seek professional support. These findings suggest that when individuals hold unfavorable beliefs or doubts toward mental health care, whether directed at professionals, digital tools, or the concept of treatment itself, these evaluations act as barriers to initiation.

### Subjective Norms and Social Expectation

Social expectations influencing initiation were identified in 17 [28,60,61,65,67,69-74,76,77,79-81,84] of the 22 included studies, grouped into 3 subthemes, including stigma, cultural beliefs, and perceived social support.

#### Stigma

Stigma emerged in 16 [28,60,61,65,67,69,70,72-74,76,77,79-81,84] studies as a significant barrier to help-seeking and initial engagement with mental health support, including DMHTs. Two distinct forms were identified, societal stigma and culturally rooted stigma.

Societal stigma refers to general negative perceptions and social judgment surrounding mental illness or help-seeking, which often results in individuals feeling shame or fear of being judged, which inhibits open discussion or action toward seeking help.

Jardine et al [74] reported, “Many participants in the study revealed that they felt ashamed of their mental distress and feared the judgment of others, feelings which had previously prevented them from seeking help or even talking about their mental health.”

On the other hand, culturally rooted stigma described in several studies is often tied to *“*negative perceptions, myths, and misconceptions associated with mental illness” [60]. When discussing mental health concerns within the cultural or ethnic communities, it has been reported that *“*They start thinking there’s some sort of witchcraft involved in it, or you’re crazy basically...” [74].

However, several studies also noted the potential for online support to mitigate this barrier. Suggesting that DMHTs *“*reduce stigma for help-seeking when compared to traditional mental health programs” [61]. By providing greater anonymity, DMHTs may reduce the risk of judgment, encouraging help-seeking.

*I often go online because I know there is something wrong, but I don’t want to tell anyone in my real life for fear that they will judge me or they won’t care and I’ll just be bothering them. Online help can help me deal with my problem alone so I will not have to tell anyone*.

(A participant in the study by Pretorius et al [81])

#### Cultural Beliefs

Cultural beliefs surrounding emotional expression also contributed to negative norms about mental health. In 3 studies, participants described the unrecognition of mental health issues as *“*We believe these things [mental health] don’t exist*”* [60]. Additionally, the discouraging cultural context leads to the open expression of sadness and other difficult emotions, promoting silence or emotional suppression. This *“*emotional suppression norm” was often framed as part of a broader cultural narrative valuing stoicism or resilience. For instance, *“*Sadness and other dysphoric emotions were broadly classified as unhealthy and unwanted” [74], while another study described the influence of *“*grind culture” and *“*resiliency narratives” that pressured individuals to *“*cope with the difficult emotions they were experiencing, which usually involved keeping them in or not expressing them” [69,74].

#### Perceived Social Support

Perceived social support was identified as a facilitator of initial engagement with DMHTs in 3 studies. Supportive encouragement from family, peers, and professionals played a key role in prompting individuals to seek help.

Family support was described as particularly influential in that conversations with family, in particular a partner or spouse, were seen as the impetus for accessing the DHMS for the initial online assessment [71].

Peer influence also emerged as a meaningful factor. Hearing about others’ experiences helped reduce stigma and normalize help-seeking behavior, *“*Hearing from peers with experience of perceived mental health support allowed for emotional release” [69]. Additionally, professional encouragement could foster more favorable attitudes toward DMHTs, as seen in *“*Recommendations from professionals to mobile DMHTs helped shape a more positive attitude and intention to seek support” [77].

#### Summary of Social Expectation Factors

In summary, social expectations strongly shaped initial engagement with DMHTs. Stigma, both societal and culturally rooted, was consistently reported as a barrier, preventing individuals from openly acknowledging mental distress and seeking support. Cultural beliefs that normalized emotional suppression or denied the existence of mental health issues further reinforced negative norms, restricting willingness to engage with digital support. On the other hand, perceived support from family, peers, and professionals provided encouragement, normalized help-seeking, and promoted more positive attitudes toward DMHTs. In some contexts, the anonymity of online services was perceived to reduce stigma, thereby transforming a barrier into an enabler.

### Perceptions of Control

The theme of PBC was identified in 14 [28,60,65,67,69-71,73,76-78,80,81,84] studies and comprised 4 subthemes, including autonomy and self-determination, awareness, perceived fit, and knowledge.

#### Autonomy and Self-Determination

A perceived lack of motivation and control over one’s actions was commonly reported as a barrier to DMHT engagement (n=5). Some participants found that digital tools offered a less intimidating alternative to traditional services, supporting greater autonomy in their help-seeking process, *“*Just turn your computer on and do it that way, it’s not quite as difficult to get yourself to do it” [74]. However, studies also highlighted that when individuals experience mental distress, initiating any form of support, including digital ones, can feel effortful and overwhelming, *“*My mental state has deteriorated. I just have no determination or willpower to do anything” [70].

#### Awareness

Lack of awareness of available DMHTs emerged as a significant barrier in 7 studies. Many individuals were unfamiliar with the existence of such tools, limiting their opportunity to initiate engagement, *“*Over half of participants did not know that mental health websites and apps existed” [78] and *“*Many young [people] don’t know that these platforms are there. So, [when] they get problems, they don’t know where to run to” [60].

#### Perceived Fit

Perceived relevance and personal alignment with the features, tone, and content of the DMHT were central to participants’ willingness to initiate use. In 8 [28,60,65,67,71,77,78,84] studies, participants emphasized that they would only consider engaging with a digital service if it felt appropriate to their needs, “Participants shared they would only seek help if the service felt ’relevant’ and ‘practical.’*”* [67].

However, determining which tool was best suited to their circumstances was often challenging. This was particularly evident among culturally diverse users, *“*Participants, who were yet to enroll in treatment, expressed doubts about whether recommended treatment options would be helpful for them*”* [28] as when scanning through the structure and content provided in the targeted DMHT, there is a *“*lack of culturally responsive treatment options and lack of diverse representation within DMHI surface content” [28].

#### Knowledge

Knowledge gaps, both about mental health and how to access and navigate digital tools, were noted in 7 studies as key impediments. These included limited mental health literacy, digital navigation skills, and uncertainty about when and how to seek help, *“*Being unsure what to search and look for online instead of searching for hours for a website that I am comfortable with” [81].

Additionally, the lack of exposure to digital mental health interventions was also evident in specific populations, *“*Egyptian students were unfamiliar with e-mental health functionalities and technical approaches” [78].

#### Summary of Perceptions of Control Factors

In summary, a lack of autonomy, motivation, and self-determination often acted as barriers, particularly when mental distress reduced the capacity to take even minimal action. Conversely, the relative ease and less intimidating nature of digital platforms could foster greater autonomy and support self-directed help-seeking.

Awareness of available tools was another critical determinant; many individuals were simply unaware of DMHT options, restricting their opportunity for engagement. Perceived fit further shaped decisions to initiate use, as participants sought services that felt personally relevant, culturally responsive, and practically aligned with their needs. Where fit was lacking, doubts and disengagement were reported. Finally, gaps in knowledge, including limited mental health literacy and uncertainty about how to navigate digital platforms, emerged as substantial barriers, impacting both initiation and continued engagement.

### Content Design and Framing

Content design and framing factors that were identified in 15 [28,60,61,65,68,70,71,73-76,78-80,83] out of the 22 included studies as factors shaped users’ perceptions of tool usability and emotional connection. Participants’ feedback highlighted 4 subthemes, content delivery format, linguistic style, embedded features, and human contact options (Table 5).

**Table 5.** Technical and logistical access considerations in digital mental health tool (DMHT) use.

Theme and subtheme	Example quote	Value, n (%)
Technical and logistical access		20 (91)
Ease of use and navigation	“Positive notes about mobile health interventions included accessibility and convenience.” [80] “Ease of use was critical; the interface needed to be simple, interactive, and easy to navigate.” [61]	4 (19)
Technical difficulties	“Broken links and disorganized flow discouraged program use and reduced return rates.” [70] "Some participants commented that technology-related factors, such as not having access to a personal computer or experiencing internet issues, could also be a barrier to engagement among students.” [73]	7 (32)
Cost and financial	"In total, 14 % of participants also stated that Cope Notes was ’too expensive’ as follows: 'I‘m not sure I would pay a month for it…when life was crazy.’" [76] “High DMH app costs and lack of payment options were major barriers for young people.” [60]	7 (32)
Transportation and geography	“I live in a rural area. there’s nothing here. You have to drive for ages.” [71] “Flexibility regarding location; Increased access for people in rural areas/living abroad.” [83]	4 (18)
Time flexibility	“'you don’t know when your emotional distress may arise, it may pop up anytime.so online resources could be a timely support.'" [67] “Access was independent of time and place; no appointments needed.” [83]	8 (36)
Privacy and confidentiality	“E-mental health can afford confidential and anonymous information and support.” [75] “Participants felt vulnerable due to uncertainty about who responds and data security.” [60]	15 (68)

#### Content Delivery Format

Participants across 5 studies underscored the importance of the delivery format in shaping their engagement. Interactive and visually dynamic formats were generally preferred over static, text-heavy approaches.

Video-based content was especially favored, with participants describing it as more accessible and engaging compared to text.

*When asked about how participants wanted the information on the EMH platform to be delivered, most participants suggested videos explaining mental health topics.*

(As reported by Mamdouh et al [78])

*“*Videos were viewed favorably; students found them easier to engage with than text-based content*”* [73].

In contrast, tools relying primarily on static formats such as SMS text messaging were perceived as limiting due to their lack of interactivity and nonverbal cues, *“*You can’t read facial expressions*”* [79].

#### Linguistic Style

Seven [28,65,70,71,73,75,76] studies explored participants’ perceptions of the linguistic style used in DMHTs. Simplicity and clarity of language were highly valued. Participants reported that straightforward content helped them remain engaged, *“*Straightforward, and it was just easy to listen to. It wasn’t like a pain” [73]. Conversely, dense or overly text-heavy content was described as a barrier, *“*Programs were too text-heavy, which deterred participation and caused users to lose interest” [70].

Emotional tone was also important. Three studies identified that warm, strength-based, and destigmatizing language could enhance the user experience and create a welcoming atmosphere, such as an *“*Overall bright and user-friendly atmosphere” [76] or a *“*Strengths-based lens and use of destigmatizing language to emphasize SMHI benefits versus deficit lens” [28]. However, an off-putting or cold tone risked disengagement, *“*Of the 14 users, 6 found that the tone of some of the texts seemed more off-putting rather than encouraging” [65].

#### Embedded Features

Six [65,68,71,73,76,80] studies reported that certain embedded features enhanced users’ interest in initiating and continuing use of DMHTs. Self-monitoring quizzes were most commonly highlighted (n=3; [71,73,78]), with participants noting their role in fostering emotional awareness and motivating further help-seeking, *“*It helped me understand how I was feeling” [73] and *“*Aided next steps in their mental health treatment journey” [71]. Data tracking features were also valued for providing personal insights, such as *“*Identify changes in mood over time” [69] and *“*I can see if there’s a good trend... maybe I should keep doing this... it was just nice to be self-aware of what was going on” [80].

#### Human Contact Options

A strong desire for human connection was reported in 11 [28,60,61,65,71,73-75,79,80,83] studies, and 2 distinct types of contact emerged: peer interaction and access to professional support.

Eight [28,60,61,65,73,75,80,83] studies indicated participants’ preference for peer-to-peer interaction features. Connecting with others who shared similar experiences helped users feel less isolated and more validated, *“*They were not alone” [73] and *“*Awareness of the shared experience of others was suggested as a facilitator of further help-seeking” [75].

Six [71,74,75,79,80,83] studies documented participants’ desire for optional access to human professionals. While digital tools were appreciated for their accessibility, many users noted the lack of empathy and personal accountability that human interaction provides, *“*Apps can be ignored. Apps can be deleted. Apps aren’t people... People would hold you accountable, people can relate to you” [80]. *“*Digital tools can lack the sense of empathy and understanding” [83].

#### Summary of Content Design and Framing Factors

In summary, the design and framing of content influenced participants’ perceptions of tool usability and their emotional connection to DMHTs. Interactive and visually dynamic delivery formats, particularly video-based content, were generally facilitators of engagement, while static, text-heavy, or SMS text messaging–based approaches were often perceived as barriers. Linguistic style played a dual role; clear, simple, and destigmatizing language fostered a welcoming user experience, whereas dense text and a cold or discouraging tone risked disengagement.

Embedded features such as self-monitoring quizzes and mood-tracking tools were valued for increasing self-awareness and motivating continued use, though the absence of meaningful interactive features could act as a limitation. Additionally, options for human contact emerged as a strong facilitator, with both peer interaction and access to professionals helping to reduce isolation and provide accountability; however, their absence highlighted a key shortcoming of fully digital interventions.

### Technical and Logistical Access

Technical and logistical access emerged as a major factor influencing individuals’ initiation and engagement with DMHTs, with relevant barriers and facilitators reported in 20 [28,60,61,65,67-71,73-83] out of the 22 included studies. This theme was further categorized into 6 subthemes, including ease of use and navigation, technical difficulties, cost and financial accessibility, transportation and geography, time flexibility, and privacy and confidentiality.

#### Ease of Use and Navigation

Participants frequently emphasized the importance of usability, highlighting that easy navigation was a key factor in shaping their experience with DMHTs. In 4 [61,73,80,83] studies, users provided positive feedback regarding the simplicity of tool interfaces across different DMHT formats. For instance, one study noted, “Positive notes about mobile health interventions included accessibility and convenience” [80]. Such design features were seen as contributing to initial engagement and continued use [61].

#### Technical Difficulties

Technical challenges were reported in 7 [60,70,73,76-78,83] studies and were found to hinder both initiation and ongoing engagement. These challenges appeared in 2 distinct forms. First, in 3 [70,76,78] studies, users encountered issues such as functional glitches and broken links, which disrupted the experience and discouraged further engagement. Second, in the remaining 4 [60,73,77,83] studies, structural access issues, such as lack of a personal digital device or unstable internet access, were identified as barriers, *“*Some participants commented that technology-related factors, such as not having access to a personal computer or experiencing internet issues, could also be a barrier to engagement among students” [73].

#### Cost and Financial Accessibility

Financial considerations were highlighted in 7 [28,60,61,65,69,81,83] studies. While participants in 2 [81,83] studies viewed DMHTs as cost-effective alternatives to traditional in-person services, 5 [28,60,61,65,69] studies reported concerns about the affordability of subscription-based digital tools. As one participant remarked, *“*Too expensive, I’m not sure I would pay $10 a month for it… when life was crazy” [65]. Additionally, a study raised concerns about digital payment access, particularly for youth, *“*It was noted that most apps require credit or debit cards to effect payment, yet most YP do not possess them” [60].

#### Transportation and Geography

Transportation and geographic accessibility were discussed in 4 [60,69,71,83] studies. Three [69,71,83] studies suggested that DMHTs offered increased flexibility and reach for individuals in rural or remote areas, *“*Increased access for people in rural areas/living abroad” [83]. However, one study pointed out that digital delivery does not entirely overcome geographic disparities, especially where internet infrastructure is weak. Mwaka et al [60] reported that “many rural areas still have limited internet connectivity.”

*Then, also accessibility is the network issue. Whereas we have a high percentage of internet penetration in Uganda, we keep on increasing but in terms of the network in most of the places especially rural settings, it is very poor. It is a struggle to get a network in these places.*

(Quote from a face-to-face focus group discussion in the study by Mwaka et al [60])

#### Time Flexibility

The flexibility to engage with services at one’s own pace and schedule was reported as a major facilitator in 8 [60,65,67,68,70,73,80,83] studies. Traditional services were described as time-consuming and rigid, *“*Lack of time or an inability to prioritize use was the largest barrier to program use” [70]. In contrast, DMHTs offered on-demand support, with one study highlighting, “Access was independent of time and place; no appointments needed” [83]. This autonomy was especially valued among individuals with demanding schedules or limited availability.

#### Privacy and Confidentiality

Privacy concerns were one of the most frequently discussed factors, raised in 15 [28,60,61,65,67,69-71,75,77-79,81-83] studies. Participants expressed apprehension about data security and the potential visibility of their personal information, particularly when services were affiliated with organizations, *“*Some respondents reported feeling vulnerable whenever they use online mental health resources because they are uncertain of who is responding to their problems, and the security of their personal information” [60], concerns were expressed about whether data might be visible to the organization [61,67].

To address such concerns, several tools incorporated features to enhance perceived privacy, such as anonymous user interaction. This was not only well received but also regarded as a central motivator for engagement, *“*Maintaining anonymity was viewed as an important motivator not only among participants who had decided to only complete online assessment but also among those who had decided to take up treatment too” [71]. In 7 [67,69,71,75,81-83] studies, anonymity and confidentiality were highlighted as benefits of digital delivery, *“*E-mental health can afford confidential and anonymous information and support” [75].

#### Summary of Content Design and Framing Factors

In summary, usability and simple navigation could serve as strong facilitators, while technical glitches, broken links, or lack of digital devices and reliable internet often acted as significant barriers. Cost-related issues presented a mixed picture, with some participants valuing digital tools as affordable alternatives to traditional care, whereas others perceived subscription fees and digital payment requirements as prohibitive. Similarly, geographic and transportation considerations highlighted that DMHTs could expand access for those in rural or remote settings, yet persistent infrastructural limitations, such as poor connectivity, undermined this benefit.

Flexibility in time and pace of engagement was one of the most consistent facilitators, particularly for individuals balancing multiple responsibilities, contrasting with the rigid demands of face-to-face services. Privacy and confidentiality concerns were frequently identified as barriers, especially regarding data security and organizational visibility of information. However, when anonymity and confidentiality were assured, these features strongly motivated engagement. Thus, factors within this theme often operated dually, with ease of use, cost, geographic reach, and privacy functioning as either barriers or facilitators depending on the specific design and context of implementation.

## Discussion

### Principal Findings

**Figure 2.**
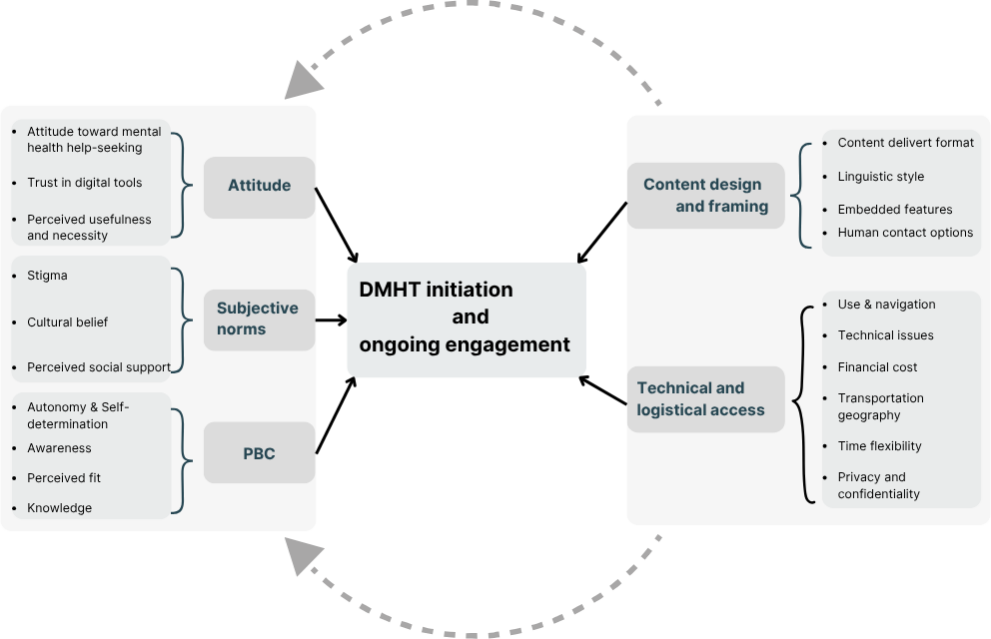
Visual summary of factors influencing engagement with digital mental health tools (DMHTs). PBC: perceived behavioral control.

This review synthesized evidence from 22 qualitative and mixed methods studies on DMHTs. Findings clustered into 2 domains aligned with the review questions (Figure 1). The first comprised the TPB: attitudes (individual evaluation), subjective norms, and PBC. The second encompassed design and access features, including cost, time flexibility, connectivity, content design, and privacy (Figure 2). These domains are interdependent: design and access conditions frequently shape the same pathways specified by the TPB, especially PBC. Across studies, the same feature often functioned as either a barrier or a facilitator, depending on users’ contexts, resources, and goals, underscoring the need for context-sensitive implementation.

### RQ1. TPB-Related Factors

Across the included studies, factors aligned with the TPB were consistently reported as important influences on the initiation and engagement with DMHTs. In this review, elements classified under subjective norms were discussed most often, followed by PBC and attitudes. This pattern mirrors findings from prior work on mental health help seeking that applied TPB, where subjective norms were the most frequently reported significant predictor, with attitudes and PBC also showing consistent associations [41].

#### Barriers Linked to Attitudes, Subjective Norms, and PBC

A set of recurring barriers emerged across the studies. First, negative attitudes toward mental health care and toward digital tools were often described. Participants in several studies expressed reluctance to identify as needing support, noting that help seeking for mental health concerns can be viewed as a sign of personal weakness, which reduced willingness to initiate use of any service, including digital options [60,69]. These observations align with prior TPB-based reviews showing that a person’s global evaluation of help-seeking is related to intention formation [41]. Specific skepticism about the usefulness, trustworthiness, or relevance of digital tools also appeared as an attitude-related deterrent to starting use [74].

Second, belief-based rejection of support functioned as a barrier. Across studies, some participants endorsed beliefs that emotional distress should be managed on one’s own or through alternative strategies such as alcohol or other substances, which reduced the perceived need for formal or digital support [85,86]. These beliefs map onto attitudes within TPB but also constrain PBC when self-reliance is framed as the only acceptable option.

Additionally, social pressures to project toughness, denying the existence of mental health conditions, and beliefs that dysphoric emotions should be suppressed or are not legitimate indicators of a need for care [60,69,74]. In our sample, these influences appeared particularly prominent in studies conducted in settings outside Western contexts. Among the 6 studies [61,62,68,70,77,79] included in this review that are conducted in non-Western countries, 5 reported stigmas as a salient deterrent to initiating digital tool use. These counts should be interpreted with caution given the qualitative nature of the synthesis and the small numbers involved. Even so, this pattern is consistent with earlier evidence that social evaluation and normative pressures have a strong bearing on help-seeking in collectivist contexts where interdependence and social approval are highly valued [21,41,44].

Knowledge gaps about DMHTs emerged as another barrier to engagement. Across the included studies, participants reported either not knowing that digital platforms for mental health support existed or, even when aware of them, not knowing how to search for and identify an appropriate option [60,81]. In settings where digital health is relatively new and not yet widely promoted, unfamiliarity with platform functions and technical approaches further limited use [49,78]. With the growing number of mental well-being tools, independent rating systems, and research that assess platforms against professional standards, an evidence base and the degree of appropriateness to users’ needs remain at an early stage [87,88]. In addition, educational elements such as basic mental health information have not always been central to implementation strategies, leaving many with a limited understanding of what digital tools can do and how they may help, which dampened initial interest [89]. Unlike traditional care pathways in which clinicians often initiate and guide help seeking, digital tools frequently require users to initiate use themselves and to navigate on their own. Field professionals have raised concerns that insufficient guidance during early encounters can undermine perceived benefit and lead users to judge tools as ineffective, thereby reducing the likelihood of future use [87,90].

#### Facilitators Linked to Attitudes, Subjective Norms, and PBC

Perceived social support from peers, family, and professionals emerged as a consistent facilitator through studies. Encouragement from close others and recommendations by peers or clinicians with similar lived experience normalized help-seeking and reduced concerns about judgment, thereby shifting subjective norms in a favorable direction [69,71,77]. In line with this pattern, studies argue that significant others, including family, friends, and colleagues, play an important role in shaping beliefs about help seeking [50]. Similarly, a study also suggests that opportunities to connect with others who had faced similar challenges created a sense of normalization and belonging, which increased willingness to try and persist with digital tools [91]. However, evidence also points to important boundary conditions. A review study of help-seeking among Filipinos illustrates a contradictory role of social networks; in some contexts, the presence of supportive friends and family may discourage formal help-seeking because informal support is viewed as sufficient or protective, dampening perceived need [92].

Perceived fit functions as a clear facilitator across settings and populations. Participants reported greater willingness to initiate and continue use when tools felt relevant and practical, and when content reflected their life circumstances [65,67]. Cultural and linguistic responsiveness, relatable examples, and alignment with users’ values enhanced a sense of personal relevance and trust, which strengthened favorable attitudes and intentions [28,73,84]. Related evidence indicates that culturally attuned design and communication from providers reduce uncertainty about suitability and promote uptake [12]. In low-resource settings, young people emphasized grounding design in local understandings of mental health and common causes of distress, which improved perceived usefulness [60]. In TPB terms, perceived fit supports intention by reinforcing positive evaluations and increasing PBC when language, features, and examples are familiar and easy to apply [41]. Alternatively, when fit is poor, the same pathways may operate in reverse and discourage engagement.

#### Factors That Can Operate as Both Barriers and Facilitators Across Contexts

Stigma was the most frequently discussed of the dual-role factors that acted in both directions depending on the context, indicating wide recognition of its relevance to the uptake of DMHTs. Prior syntheses have identified stigma and embarrassment as key obstacles to help-seeking, engagement with care, and adherence to treatment [93,94]. In several studies in our sample, users described the weight of social evaluation and the broader idea that “not being mentally well” attracts judgment, which discouraged help seeking irrespective of delivery mode [70,74]. At the same time, some participants viewed digital options as less stigmatizing than conventional services, since greater privacy and a degree of anonymity could reduce fear of negative reactions and make initial contact feel safer [15,95]. Importantly, self-stigma may still limit action even when anonymity is available; individuals who internalize negative beliefs about treatment can be reluctant to seek information online and to approach in-person services [72,77,96]. Within the TPB, these patterns suggest that stigma can depress intentions through unfavorable attitudes and through perceived social pressure, yet privacy features and supportive messaging can shift subjective norms in a favorable direction when users feel protected and accepted.

### RQ2. DMHTs Design- and Access-Related Factors

Across the included studies, features of DMHTs related to design and access shaped both the decision to initiate use and the capacity to persist. The most consistent patterns concerned costs, time flexibility and immediacy, geographic and logistical accessibility, content design and framing, and privacy and confidentiality

#### Barriers Linked to Design and Access

Although digital delivery is often promoted as a lower-cost alternative to in-person care, financial barriers remained salient. Prior work on in-person care has shown that treatment fees, travel, and lost wages constrain help-seeking [86,97-99]. In principle, DMHTs can reduce some of these outlays by offering low-cost alternatives [16]. In our review, several studies reflected this potential, noting comparatively low monetary cost and perceived affordability [69,81,83].

However, most studies that discussed finances still framed cost as a barrier to initiation and sustained use. Even when programs are free at the point of use, participants may need a modern device, stable data plans, and paid features or add-ons embedded within applications [28,61,65]. Payment systems that assume credit or debit cards further exclude users in low-resource settings [60]. Critically, these costs are unevenly distributed and can reproduce existing inequities, such that a tool that lowers average cost can still be financially inaccessible to those with the least capacity to pay [15].

#### Facilitators Linked to Design and Access

Time flexibility emerged as a decisive facilitator. Participants valued round-the-clock availability and the ability to access support when distress peaks outside standard clinic hours [49,61,81]. Short, modular activities were seen as easy to fit into everyday routines, which reduced friction and strengthened PBC [68]. This immediacy aligns with on-demand patterns of help-seeking and can transform fleeting motivation into timely action [100].

DMHTs also reduced travel demands and scheduling constraints associated with in-person services, a benefit that is especially meaningful for people in rural or remote regions [101,102]. In our review, most studies that discussed geography reported that digital delivery enabled counseling or self-help beyond local service catchments, including for people living abroad [69,71,83]. The capacity to access support more immediately and with fewer logistical constraints may enhance users’ perceived sense of control over when and how they seek help.

Across a range of design formats, participants preferred interactive and visually engaging content over static text. Video explanations and audio options were consistently reported as more accessible and engaging, particularly for complex topics [73]. Language style mattered as well. Warm, straightforward, and destigmatizing language lowered the emotional burden of entry, created a more user-friendly environment, and encouraged continued interaction [28,65]. These patterns echo prior reviews that link satisfaction with content presentation to higher engagement and note preferences for a supportive, not judgmental tone, and multimodal delivery [15].

#### Factors That Can Operate as Both Barriers and Facilitators Across Contexts

Privacy and confidentiality concerns were pervasive, but their effects were bidirectional. On the one hand, participants expressed uncertainty about data storage, data sharing, and the safety of sensitive information, especially when tools were provided by an employer or other affiliated organization [60,67,70]. The degree of transparency around what data are collected, how they are stored, and who may access them could influence the development of trust between users and DMH service providers [67]. From a TPB-informed perspective, this trust may in turn shape users’ attitudes toward the tool, contributing to either more favorable or less favorable evaluations of engagement.

On the other hand, some studies found that digital delivery increased perceived privacy by allowing anonymous or low-exposure contact, which made participants more willing to disclose and to engage compared with face-to-face encounters [71,75]. Prior work has similarly noted that anonymity can be especially enabling for those who fear stigma [93].

### Implications

This review synthesized evidence on DMHTs to identify common factors that shape users’ engagement and experience. Translating these findings into practice suggests a 2-track strategy. First, strengthen willingness to seek support by addressing attitudes, social expectations, and PBC as outlined in the TPB. Second, design and deliver services that reduce effort at every step while building trust through clear communication and dependable safeguards.

Policy has a central role in this agenda. Reducing stigma and fear around help-seeking, raising awareness of available services, including digital options, and improving equitable access are critical. This includes addressing data costs, device constraints, and connectivity, which can suppress uptake among people with fewer resources. Policies provide broader efforts that normalize help-seeking among family members and the general public can build supportive norms and produce cumulative benefits over time [50,67]. Schools are another important setting. Integrating age-appropriate mental health knowledge and help-seeking skills into the curriculum can foster open attitudes among young people and improve early access to support [103]. Structural conditions, such as data costs, unstable internet access, and limited device availability, constrain use of DMHTs, particularly in low-resource settings [60]. Without deliberate mitigation, DMHTs can unintentionally widen inequities. The Digital Health Equity Framework highlights how poverty and restricted digital access can contribute to poorer health outcomes [104]. Equity considerations should therefore guide policy, commissioning, and product development to ensure digital health benefits are realized across diverse communities.

Developers can use these findings to guide product choices that support engagement. Usability needs to be tested and improved for the intended audience, including simple navigation, stable performance, and timely technical assistance. Content should be tailored and presented in a warm and supportive manner that respects diverse experiences and preferences. Trust requires more than privacy policies, and users could benefit from plain language explanations of what data are collected, how they are protected, and who can access them. Co-design with intended user groups, including people from culturally diverse communities and rural areas, can help ensure that features align with real needs and contexts [78].

Service providers can apply these insights when matching clients to tools. A key finding across the studies is that perceived fit strongly influences whether people start and continue with self-care DMHTs. Fit includes personal relevance of goals, cultural and linguistic appropriateness, and practical alignment with daily routines and constraints. Providers should assess these elements during intake and review and support users to select tools with content and features that feel suitable and relatable.

### Limitations and Recommendations for Future Research

Several limitations should be considered when interpreting the findings. First, although many included studies involved participants from diverse ethnic backgrounds, most primary studies did not analyze experiences separately by cultural group. As a result, it limited the conclusions this study could draw specific to particular cultural contexts. Future reviews should prioritize designs and search strategies that enable analysis by culture, language, and migration status and should report subgroup findings in a clear and consistent manner.

Second, most of the studies reviewed were conducted in resource-limited countries that have strong and stable economies and greater access to health care and education [105]. In this review, 82% of the included studies came from such settings. Our understanding of experiences in lower-resource contexts remains limited. Factors linked to availability and access, such as connectivity, data costs, device access, and local service integration, are likely to vary across settings and may shape both uptake and sustained use in different ways. More research from low- and middle-income countries and from rural or remote communities is needed to test how these contextual factors influence engagement.

Third, there was marked inconsistency in how studies measured satisfaction, engagement, and continued use. This problem has been observed in earlier reviews of digital mental health interventions [15,106]. Inconsistent definitions and instruments reduce comparability across studies, limit the ability to detect patterns, and make it difficult to combine results. They can also bias conclusions when similar terms refer to different constructs or when short follow-up periods are used to infer sustained engagement.

This review focused primarily on user experience; most included studies used qualitative designs, such as semistructured interviews and face-to-face group discussions. These methods are well suited to explore perceptions and context, but they limit statistical generalization. The heterogeneity of designs, samples, and analytic approaches further reduced the scope for quantitative synthesis. Future quantitative studies that estimate the statistical impact of the association between specific factors and engagement with DMHTs would add value for policy and practice.

A further limitation of this review is that the included studies were primarily based on one-off interviews and survey data, with little evidence from other data collection approaches, such as ecological momentary assessment, diary methods, or broader longitudinal designs. Although these approaches were not explicitly excluded by the eligibility criteria, studies using them may have been underrepresented in the available literature within this field. The relative absence of these methods means that the present synthesis is more reflective of retrospective and cross-sectional accounts of user experience and may therefore be less able to capture fluctuations in engagement, moment-to-moment barriers to use, and how attitudes or behaviors change over time. As engagement with DMHTs is often dynamic and shaped by everyday circumstances, future research would benefit from incorporating data collection approaches that are able to capture user experiences as they unfold in context. Such approaches may provide a richer understanding of when, how, and why barriers and facilitators emerge across the course of use.

Finally, stigma emerged in participants’ accounts as a factor shaping both general help seeking and engagement with DMHTs. Prior work suggests that not all stigma constructs operate equally; for example, negative help-seeking attitudes and personal stigma show stronger and more consistent associations with reduced support seeking than some other stigma types [2]. In this review, we considered attitudes and general stigma, but differential effects of distinct stigma dimensions (eg, self-stigma, perceived public stigma, and anticipated stigma) on DMHT uptake and continuation were not examined. Future research should investigate how specific stigma types, and their interaction with cultural and contextual factors, influence adoption and ongoing use.

### Conclusion

This review shows that engagement with DMHTs is not driven by design alone or by beliefs in isolation. Rather, attitudes, social expectations, and perceived behavioral control interact with the practical realities of cost, connectivity, device access, time flexibility, content design, and privacy. When design and access conditions support feasibility and trust, they amplify the same TPB pathways that foster intention and continued use. When they do not, they undermine them, particularly by eroding perceived control.

For policy and practice, a dual approach is suggested: (1) strengthen willingness to seek support by addressing attitudes and norms and by reducing stigma and (2) provide support to the user journey through reliable connectivity, affordable data and devices, usable interfaces, culturally and linguistically appropriate content, and plain-language data protections.

The evidence base remains constrained by concentration in high-income settings, limited cultural subgroup analyses, and inconsistent engagement measures. Future work should include studies in lower-resource and rural contexts, adopt common, theory-informed metrics with appropriate timeframes, and examine how distinct stigma dimensions influence both adoption and continuation. Advancing along these lines will enable DMHTs to deliver equitable, trustworthy, and sustained benefits at scale.

## Supplementary material

10.2196/88731 Multimedia Appendix 1 Search strategy.

10.2196/88731 Multimedia Appendix 2 Risk of bias assessment of the included studies for quality appraisal.

10.2196/88731 Multimedia Appendix 3 Raw excerpts and code labels.

10.2196/88731 Multimedia Appendix 4 General descriptive features of include studies.

10.2196/88731 Checklist 1 PRISMA checklist.

## Abbreviations

DMHT: digital mental health tool
PBC: perceived behavioral control
PICO: Population, Intervention, Comparator, and Outcome framework
PICOC: Population, Intervention, Comparator, Outcome, and Context
PICOS: Population, Intervention, Comparator, Outcome, and Study design
PRESS: Peer Review of Electronic Search Strategies
PRISMA: Preferred Reporting Items for Systematic Reviews and Meta-Analyses
QuADS: quality assessment with diverse studies
TPB: theory of planned behavior

## References

[1] (2025). Mental disorders. World Health Organization.

[2] Schnyder N, Panczak R, Groth N, Schultze-Lutter F (2017). Association between mental health-related stigma and active help-seeking: systematic review and meta-analysis. Br J Psychiatry.

[3] Mann K, Tesfamichael S, Rimes KA (2024). The impact of role models on sexual minority women: a qualitative interview study. Behav Sci (Basel).

[4] Chiu MYL, Yang X, Wong HT, Li JH (2015). The mediating effect of affective stigma between face concern and general mental health - the case of Chinese caregivers of children with intellectual disability. Res Dev Disabil.

[5] George N, Lee AH, Gonzalez-Gaspar J, Kwan MY (2024). A systematic review of barriers and facilitators impacting the utilization of mental health services among asian American youth. Evid Based Pract Child Adolesc Ment Health.

[6] Kim SB, Lee YJ (2022). Factors associated with mental health help-seeking among Asian Americans: a systematic review. J Racial Ethn Health Disparities.

[7] Kirkbride JB, Anglin DM, Colman I (2024). The social determinants of mental health and disorder: evidence, prevention and recommendations. World Psychiatry.

[8] Evans-Lacko S, Hahn JS, Peter LJ, Schomerus G (2022). The impact of digital interventions on help-seeking behaviour for mental health problems: a systematic literature review. Curr Opin Psychiatry.

[9] Rathbone AL, Prescott J (2017). The use of mobile apps and SMS messaging as physical and mental health interventions: systematic review. J Med Internet Res.

[10] Wang Q, Zhang W, An S (2023). A systematic review and meta-analysis of Internet-based self-help interventions for mental health among adolescents and college students. Internet Interv.

[11] (2022). World mental health report: transforming mental health for all. World Health Organization.

[12] Zhao R, Amanvermez Y, Pei J (2025). Research review: help-seeking intentions, behaviors, and barriers in college students - a systematic review and meta-analysis. J Child Psychol Psychiatry.

[13] Mahoney A, Li I, Haskelberg H, Millard M, Newby JM (2021). The uptake and effectiveness of online cognitive behaviour therapy for symptoms of anxiety and depression during COVID-19. J Affect Disord.

[14] Marshall JM, Dunstan DA, Bartik W (2020). The role of digital mental health resources to treat trauma symptoms in Australia during COVID-19. Psychol Trauma.

[15] Borghouts J, Eikey E, Mark G (2021). Barriers to and facilitators of user engagement with digital mental health interventions: systematic review. J Med Internet Res.

[16] Johnson JA, Sanghvi P, Mehrotra S (2022). Technology-based interventions to improve help-seeking for mental health concerns: a systematic review. Indian J Psychol Med.

[17] Ekoh PC, Nnadi F, Nwabineli H (2024). Barriers to the use of mental health services amongst men in Nigeria and the potential of digital mental health support. Adv Ment Health.

[18] Mahalik JR, Di Bianca M (2021). Help-seeking for depression as a stigmatized threat to masculinity. Prof Psychol Res Pr.

[19] Carter H, Araya R, Anjur K, Deng D, Naslund JA (2021). The emergence of digital mental health in low-income and middle-income countries: a review of recent advances and implications for the treatment and prevention of mental disorders. J Psychiatr Res.

[20] Naslund JA, Aschbrenner KA, Kim SJ (2017). Health behavior models for informing digital technology interventions for individuals with mental illness. Psychiatr Rehabil J.

[21] Shi W, Shen Z, Wang S, Hall BJ (2020). Barriers to professional mental health help-seeking among Chinese adults: a systematic review. Front Psychiatry.

[22] Taylor-Rodgers E, Batterham PJ (2014). Evaluation of an online psychoeducation intervention to promote mental health help seeking attitudes and intentions among young adults: randomised controlled trial. J Affect Disord.

[23] Choi I, Mestroni G, Hunt C, Glozier N (2023). Personalized help-seeking web application for Chinese-speaking international university students: development and usability study. JMIR Form Res.

[24] Gan DZQ, McGillivray L, Han J, Christensen H, Torok M (2021). Effect of engagement with digital interventions on mental health outcomes: a systematic review and meta-analysis. Front Digit Health.

[25] Rushton K, Ardern K, Hopkin E (2020). “I didn’t know what to expect”: exploring patient perspectives to identify targets for change to improve telephone-delivered psychological interventions. BMC Psychiatry.

[26] Pywell J, Vijaykumar S, Dodd A, Coventry L (2020). Barriers to older adults’ uptake of mobile-based mental health interventions. Digit Health.

[27] Thomas J, Barraket J, Wilson CK (2020). Measuring Australia’s digital divide: the Australian digital inclusion index 2020 (report for Telstra).

[28] Kodish T, Schueller SM, Lau AS (2023). Barriers and strategies to improve digital mental health intervention uptake among college students of color: a modified Delphi study. J Behav Cogn Ther.

[29] Albanese AM, Smith BE, Geller PA (2023). To guide or to self-guide?: predictors of preferring a guided introduction to digital resources that promote postpartum mental health. Psychiatry International.

[30] Lattie EG, Stiles-Shields C, Graham AK (2022). An overview of and recommendations for more accessible digital mental health services. Nat Rev Psychol.

[31] Balcombe L, De Leo D (2023). Evaluation of the use of digital mental health platforms and interventions: scoping review. Int J Environ Res Public Health.

[32] Philippe TJ, Sikder N, Jackson A (2022). Digital health interventions for delivery of mental health care: systematic and comprehensive meta-review. JMIR Ment Health.

[33] Werntz A, Amado S, Jasman M, Ervin A, Rhodes JE (2023). Providing human support for the use of digital mental health interventions: systematic meta-review. J Med Internet Res.

[34] Lehtimaki S, Martic J, Wahl B, Foster KT, Schwalbe N (2021). Evidence on digital mental health interventions for adolescents and young people: systematic overview. JMIR Ment Health.

[35] Bifftu BB, Thomas SJ, Win KT (2025). Users’ positive attitudes, perceived usefulness, and intentions to use digital mental health interventions: a systematic literature review and meta-analysis. Comput Biol Med.

[36] Bohon LM, Cotter KA, Kravitz RL, Cello PC, Fernandez Y Garcia E (2016). The theory of planned behavior as it predicts potential intention to seek mental health services for depression among college students. J Am Coll Health.

[37] Engelhardt EC, Bicknell G, Oliver M, Flaherty C, Line K, King E (2023). Theory of planned behavior and active duty air force members’ mental health help-seeking. Mil Med.

[38] Ajzen I (2011). The theory of planned behaviour: reactions and reflections. Psychol Health.

[39] Ajzen I, Fishbein M (1980). Understanding Attitudes and Predicting Social Behavior.

[40] Conner M, Tenenbaum G, Eklund RC, Boiangin N Handbook of Sport Psychology: Social Perspectives, Cognition, and Applications.

[41] Adams C, Gringart E, Strobel N (2022). Explaining adults’ mental health help-seeking through the lens of the theory of planned behavior: a scoping review. Syst Rev.

[42] Conner M, Heywood-everett S (1998). Addressing mental health problems with the theory of planned behaviour. Psychol Health Med.

[43] Schomerus G, Matschinger H, Angermeyer MC (2009). Attitudes that determine willingness to seek psychiatric help for depression: a representative population survey applying the theory of planned behaviour. Psychol Med.

[44] Mo PKH, Mak WWS (2009). Help-seeking for mental health problems among Chinese. Soc Psychiat Epidemiol.

[45] Khatib A, Abo-Rass F, Gelkopf M (2022). Theory of planned behavior: exploring the use of digital mental health interventions in Israel. J Nerv Ment Dis.

[46] Clough B, Yousif C, Miles S, Stillerova S, Ganapathy A, Casey L (2022). Understanding client engagement in digital mental health interventions: an investigation of the eTherapy attitudes and process questionnaire. J Clin Psychol.

[47] Valenstein M, Kavanagh J, Lee T (2011). Using a pharmacy-based intervention to improve antipsychotic adherence among patients with serious mental illness. Schizophr Bull.

[48] McKibbin CL, Patterson TL, Norman G (2006). A lifestyle intervention for older schizophrenia patients with diabetes mellitus: a randomized controlled trial. Schizophr Res.

[49] Kauer SD, Mangan C, Sanci L (2014). Do online mental health services improve help-seeking for young people? A systematic review. J Med Internet Res.

[50] Mak HW, Davis JM (2014). The application of the theory of planned behavior to help-seeking intention in a Chinese society. Soc Psychiatry Psychiatr Epidemiol.

[51] Booth A, Sutton A, Clowes M, Martyn-St James M (2021). Systematic Approaches to a Successful Literature Review.

[52] Moher D, Liberati A, Tetzlaff J, Altman DG, Group P (2009). Preferred reporting items for systematic reviews and meta-analyses: the PRISMA statement. PLOS Med.

[53] Harrison R, Jones B, Gardner P, Lawton R (2021). Quality assessment with diverse studies (QuADS): an appraisal tool for methodological and reporting quality in systematic reviews of mixed- or multi-method studies. BMC Health Serv Res.

[54] McGowan J, Sampson M, Salzwedel DM, Cogo E, Foerster V, Lefebvre C (2016). PRESS peer review of electronic search strategies: 2015 guideline statement. J Clin Epidemiol.

[55] Haddaway NR, Collins AM, Coughlin D, Kirk S (2015). The role of Google Scholar in evidence rreviews and its applicability to grey literature searching. PLOS One.

[56] Eriksen MB, Frandsen TF (2018). The impact of patient, intervention, comparison, outcome (PICO) as a search strategy tool on literature search quality: a systematic review. J Med Libr Assoc.

[57] Methley AM, Campbell S, Chew-Graham C, McNally R, Cheraghi-Sohi S (2014). PICO, PICOS and SPIDER: a comparison study of specificity and sensitivity in three search tools for qualitative systematic reviews. BMC Health Serv Res.

[58] Alsahli S, Hor SY, Lam M (2023). Factors influencing the acceptance and adoption of mobile health apps by physicians during the COVID-19 pandemic: systematic review. JMIR Mhealth Uhealth.

[59] Timmermans S, Tavory I (2012). Theory construction in qualitative research: From grounded theory to abductive analysis. Sociol Theory.

[60] Mwaka ES, Bazzeketa D, Mirembe J, Emoru RD, Twimukye A, Kivumbi A (2025). Barriers to and enhancement of the utilization of digital mental health interventions in low-resource settings: perceptions of young people in Uganda. Digit HEALTH.

[61] Tan CJY, Müller AM, Rajendram P, Subramaniam M (2025). Factors affecting the adoption of mental health apps in workplaces: a qualitative study. J technol behav sci.

[62] Braun V, Clarke V (2006). Using thematic analysis in psychology. Qual Res Psychol.

[63] Ng A, Reddy M, Zalta AK, Schueller SM (2018). Veterans’ perspectives on Fitbit use in treatment for post-traumatic stress disorder: an interview study. JMIR Ment Health.

[64] Lienemann BA, Unger JB, Cruz TB, Chu KH (2017). Methods for coding tobacco-related Twitter data: a systematic review. J Med Internet Res.

[65] King SL, Lebert J, Karpisek LA, Phillips A, Neal T, Kosyluk K (2022). Characterizing user experiences with an SMS text messaging-based mHealth intervention: mixed methods study. JMIR Form Res.

[66] Sandelowski M (2001). Real qualitative researchers do not count: the use of numbers in qualitative research. Res Nurs Health.

[67] Chan K yi, Yeung N yiu, Mo P han, Yang X (2024). Common stressors, coping processes, and professional help-seeking of medical professionals in Hong Kong: a qualitative study. J Health Psychol.

[68] Cliffe B, Stokes Z, Stallard P (2023). The acceptability of a smartphone app (BlueIce) for university students who self-harm. Arch Suicide Res.

[69] Dela Cruz ENM, Marcelo RCA, Naling BYM, Ty WEG (2023). “Hello, can you hear me?”: narratives of online mental health counselling among Filipino adults during the pandemic. Couns and Psychother Res.

[70] Eccles H, Nannarone M, Lashewicz B (2021). Barriers to the sue of web-based mental health programs for preventing depression: qualitative study. JMIR Form Res.

[71] Fisher A, Corrigan E, Cross S (2024). Decision-making about uptake and engagement with digital mental health services: a qualitative exploration of service user perspectives. Clin Psychol (Aust Psychol Soc).

[72] Hoffman BD, Oppert ML, Owen M (2024). Understanding young adults’ attitudes towards using AI chatbots for psychotherapy: the role of self-stigma. Comput Hum Behav Artif Hum.

[73] Jackson HM, Gulliver A, Hasking P (2024). Exploring student preferences for implementing a digital mental health intervention in a university setting: qualitative study within a randomised controlled trial. Digit HEALTH.

[74] Jardine J, Nadal C, Robinson S, Enrique A, Hanratty M, Doherty G (2024). Between rhetoric and reality: real-world barriers to uptake and early engagement in digital mental health interventions. ACM Trans Comput-Hum Interact.

[75] Karwig G, Chambers D (2016). Annual Review of Cybertherapy and Telemedicine 2016.

[76] Kim H, Kim M, Heo J in, Lee S, Kim H, Jung D (2025). Understanding university students’ experiences on multi-domain help seeking platform “fruto”: vignettes study. https://dl.acm.org/doi/proceedings/10.1145/3706599.

[77] Levin ME, Stocke K, Pierce B, Levin C (2018). Do college students use online self-help? A survey of intentions and use of mental health resources. J College Stud Psychother.

[78] Mamdouh M, Tai AMY, Westenberg JN (2022). Egyptian students open to digital mental health care: cross-sectional survey. JMIR Form Res.

[79] McCall T, Foster M, Schwartz TA (2023). Attitudes toward seeking mental health services and mobile technology to support the management of depression among Black American women: cross-sectional survey study. J Med Internet Res.

[80] McCarthy K, Horwitz AG (2025). Attitudes and barriers to mobile mental health interventions among first-year college students: a mixed-methods study. J Am Coll Health.

[81] Pretorius C, Chambers D, Cowan B, Coyle D (2019). Young people seeking help online for mental health: cross-sectional survey study. JMIR Ment Health.

[82] Tickell A, Fonagy P, Hajdú K, Obradović S, Pilling S (2024). “Am I really the priority here?”: help-seeking experiences of university students who self-harmed. BJPsych Open.

[83] Wallin EEK, Mattsson S, Olsson EMG (2016). The preference for internet-based psychological interventions by individuals without past or current use of mental health treatment delivered online: a survey study with mixed-methods analysis. JMIR Ment Health.

[84] Wallin E, Maathz P, Parling T, Hursti T (2018). Self-stigma and the intention to seek psychological help online compared to face-to-face. J Clin Psychol.

[85] Ali K, Farrer L, Fassnacht DB, Gulliver A, Bauer S, Griffiths KM (2017). Perceived barriers and facilitators towards help-seeking for eating disorders: a systematic review. Int J Eat Disord.

[86] Brown A, Rice SM, Rickwood DJ, Parker AG (2016). Systematic review of barriers and facilitators to accessing and engaging with mental health care among at-risk young people. Asia Pac Psychiatry.

[87] Torous J, Levin ME, Ahern DK, Oser ML (2017). Cognitive behavioral mobile applications: clinical studies, marketplace overview, and research agenda. Cogn Behav Pract.

[88] Hopkins T, Kwon E, Lapins A, Gill N, Roberts A, Rogalski E (2024). Assessment of disease impact through health‐related quality of life measurement in primary progressive aphasia. Alzheimers Dement (N Y).

[89] O’Connor S, Hanlon P, O’Donnell CA, Garcia S, Glanville J, Mair FS (2016). Understanding factors affecting patient and public engagement and recruitment to digital health interventions: a systematic review of qualitative studies. BMC Med Inform Decis Mak.

[90] Pierce B, P. Twohig M, Levin ME (2016). Perspectives on the use of acceptance and commitment therapy related mobile apps: results from a survey of students and professionals. J Contextual Behav Sci.

[91] Ho TQA, Le LKD, Engel L (2025). Barriers to and facilitators of user engagement with web-based mental health interventions in young people: a systematic review. Eur Child Adolesc Psychiatry.

[92] Martinez AB, Co M, Lau J, Brown JSL (2020). Filipino help-seeking for mental health problems and associated barriers and facilitators: a systematic review. Soc Psychiatry Psychiatr Epidemiol.

[93] Gulliver A, Griffiths KM, Christensen H (2010). Perceived barriers and facilitators to mental health help-seeking in young people: a systematic review. BMC Psychiatry.

[94] Stangl AL, Earnshaw VA, Logie CH (2019). The Health Stigma and Discrimination Framework: a global, crosscutting framework to inform research, intervention development, and policy on health-related stigmas. BMC Med.

[95] Stephens-Reicher J, Metcalf A, Blanchard M, Mangan C, Burns J (2011). Reaching the hard-to-reach: how information communication technologies can reach young people at greater risk of mental health difficulties. Australas Psychiatry.

[96] Lannin DG, Vogel DL, Brenner RE, Abraham WT, Heath PJ (2016). Does self-stigma reduce the probability of seeking mental health information?. J Couns Psychol.

[97] Chandrashekar P (2018). Do mental health mobile apps work: evidence and recommendations for designing high-efficacy mental health mobile apps. Mhealth.

[98] Esponda GM, Hartman S, Qureshi O, Sadler E, Cohen A, Kakuma R (2020). Barriers and facilitators of mental health programmes in primary care in low-income and middle-income countries. Lancet Psychiatry.

[99] Kavanagh BE, Corney KB, Beks H, Williams LJ, Quirk SE, Versace VL (2023). A scoping review of the barriers and facilitators to accessing and utilising mental health services across regional, rural, and remote Australia. BMC Health Serv Res.

[100] Di Domenico SI, Ryan RM (2017). The emerging neuroscience of intrinsic motivation: a new frontier in self-determination research. Front Hum Neurosci.

[101] De Silva T, Prakash A, Yarlagadda S (2017). General practitioners’ experiences and perceptions of mild moderate depression management and factors influencing effective service delivery in rural Australian communities: a qualitative study. Int J Ment Health Syst.

[102] Liverpool S, Mota CP, Sales CMD (2020). Engaging children and young people in digital mental health interventions: systematic review of modes of delivery, facilitators, and barriers. J Med Internet Res.

[103] Goetz CJ, Mushquash CJ, Maranzan KA (2023). An integrative review of barriers and facilitators associated with mental health help seeking among Indigenous populations. Psychiatr Serv.

[104] Crawford A, Serhal E (2020). Digital health equity and COVID-19: the innovation curve cannot reinforce the social gradient of health. J Med Internet Res.

[105] Heshmati A, Kim J, Park D, Park D, Lee SH, Lee M (2015). Inequality, Inclusive Growth, and Fiscal Policy in Asia.

[106] Fleming T, Bavin L, Lucassen M, Stasiak K, Hopkins S, Merry S (2018). Beyond the trial: systematic review of real-world uptake and engagement with digital self-help interventions for depression, low mood, or anxiety. J Med Internet Res.

