# Supplementary material for: Factors Influencing the Initiation and Continued Engagement of Digital Mental Health Tools Among Adults: Theory of Planned Behavior–Informed Systematic Review
Source: JMIR Ment Health. 2026 May 15;13:e88731. doi: 10.2196/88731 (PMC13179054; doi:10.2196/88731)
Supplement: Multimedia Appendix 1 [file mental-v13-e88731-s001.docx]

**Appendix 1**

*Search Strategy*

General Search String:

("digital mental health" OR "mental health app*" OR "e-mental health" OR "online mental health" OR "tele-mental health" OR "digital intervention*" OR "mobile health" OR mHealth OR eHealth)

AND

("help-seeking" OR "service initiation" OR "access to care" OR "mental health support" OR "early engagement" OR "first contact")

AND

("barrier*" OR "facilitator*" OR "attitude*" OR "stigma" OR "trust" OR "user experience" OR "perception*" OR "acceptability" OR "cultural relevance")

Search String for PudMed:

(Mental Health Services[Title/Abstract] OR digital mental health[Title/Abstract] OR mental health app[Title/Abstract] OR e-mental health[Title/Abstract] OR online mental health[Title/Abstract] OR tele-mental health[Title/Abstract] OR mHealth[Title/Abstract] OR mobile health[Title/Abstract])

AND ("help-seeking behavior"[MeSH] OR "help-seeking"[tiab] OR "service initiation"[tiab] OR "early engagement"[tiab])

 AND (barriers[Title/Abstract] OR facilitators[Title/Abstract] OR attitude*[Title/Abstract] OR trust[Title/Abstract] OR stigma[Title/Abstract] OR cultural relevance[Title/Abstract] OR user experience[Title/Abstract])

Search String for EMBASE:

('digital mental health':ti,ab OR 'mental health app*':ti,ab OR 'e-mental health':ti,ab OR 'tele-mental health':ti,ab OR mhealth:ti,ab OR ehealth:ti,ab)

AND

('help seeking':ti,ab OR 'service initiation':ti,ab OR 'access to care':ti,ab OR 'early engagement':ti,ab)

AND

(barrier* OR facilitator* OR stigma OR 'user experience' OR trust OR 'cultural relevance')

Search String for PsycINFO:

("digital mental health" OR "mental health app*" OR "e-mental health" OR "online mental health" OR "tele-mental health" OR mHealth OR eHealth)

AND

("help-seeking" OR "service initiation" OR "early engagement" OR "access to care")

AND

("barrier*" OR "facilitator*" OR "user experience" OR "attitude*" OR "trust" OR "stigma" OR "cultural relevance")

Search String for Scopus:

(TITLE-ABS-KEY("digital mental health" OR "mental health app*" OR "e-mental health" OR "mHealth" OR "tele-mental health"))

AND

(TITLE-ABS-KEY("help-seeking" OR "service initiation" OR "early engagement"))

AND

("barrier*" OR "facilitator*" OR "attitude*" OR "stigma" OR "trust" OR "user experience" OR "cultural relevance")

Search String for Cochrane Central Register of Controlled Trials:

("digital mental health" OR "mental health app*" OR "e-mental health")  in Title Abstract Keyword

AND

("help-seeking" OR "access to care" OR "service initiation")  in Title Abstract Keyword

AND

("barriers" OR "facilitators" OR "user experience")

Search String for CINAHL Complete:

("digital mental health" OR "mental health apps" OR "e-mental health" OR "tele-mental health")

AND

("help-seeking" OR "service access" OR "service navigation")

AND

("barriers" OR "facilitators" OR "user experience" OR "stigma")

Search String for Web of Science:

("digital mental health" OR "mental health app*" OR "e-mental health" OR "mHealth")

AND ("help-seeking" OR "service initiation")

AND ("barrier*" OR "facilitator*" OR "user experience" OR "cultural relevance" OR "trust")

Search String for ACM Digital Library:

("digital mental health" OR "mental health app" OR chatbot)

AND

("help-seeking" OR "access to service" OR "service initiation")

AND

("barrier*" OR "facilitator*" OR "attitude*" OR "stigma" OR "trust" OR "user experience" OR "cultural relevance")

Search String for Google Scholar (for grey literature):

("digital mental health" AND "help-seeking" AND (barriers OR facilitators OR stigma OR "user experience")
