# Supplementary material for: Factors Influencing the Initiation and Continued Engagement of Digital Mental Health Tools Among Adults: Theory of Planned Behavior–Informed Systematic Review
Source: JMIR Ment Health. 2026 May 15;13:e88731. doi: 10.2196/88731 (PMC13179054; doi:10.2196/88731)
Supplement: Multimedia Appendix 2 [file mental-v13-e88731-s002.docx]

**Appendix 2**

*Short ID to each study*

| First Author and Year | | Study ID | |
| --- | --- | --- | --- |
| Chan et al (2024) [68] | | S01 |  |
| Cliffe et al (2023) [69] | | S02 |  |
| Dela Cruz et al (2023) [70] | | S03 |  |
| Eccles et al (2021) [71] | | S04 |  |
| Fisher et al (2024) [72] | | S05 |  |
| Hoffman et al (2024) [73] | | S06 |  |
| Jackson et al (2024) [74] | | S07 |  |
| Jardine et al (2024) [75] | | S08 |  |
| Karwig & Chamber (2016) [76] | | S09 |  |
| Kim et al (2025) [77] | | S10 |  |
| King et al (2022) [66] | | S11 |  |
| Kodish et al (2023) [28] | | S12 |  |
| Levin et al (2018) [78] | | S13 |  |
| Mamdouh et al (2022) [79] | | S14 |  |
| McCall et al (2023) [80] | | S15 |  |
| McCarthy & Horwitz (2025) [81] | | S16 |  |
| Mwaka et al (2025) [61] | | S17 |  |
| Pretorius et al (2019) [82] | | S18 |  |
| Tan et al (2025) [62] | | S19 |  |
| Tickell et al (2024) [83] | | S20 |  |
| Wallin et al (2016) [84] | | S21 |  |
| Wallin et al (2018) [85] | | S22 |  |

References:

28. Kodish T, Schueller SM, Lau AS. Barriers and strategies to improve digital mental health intervention uptake among college students of color: A modified Delphi study. Journal of Behavioral and Cognitive Therapy. Mar 2023;33(1):10-23. [doi: ]

61. Mwaka ES, Bazzeketa D, Mirembe J, Emoru RD, Twimukye A, Kivumbi A. Barriers to and enhancement of the utilization of digital mental health interventions in low-resource settings: Perceptions of young people in Uganda. Digit HEALTH. 2025;11:20552076251321698. [doi: ] [Medline: 39963503]

62. Tan CJY, Müller AM, Rajendram P, Subramaniam M. Factors Affecting the Adoption of Mental Health Apps in Workplaces: A Qualitative Study. J technol behav sci. 2025. [doi: 10.1007/s41347-025-00524-z]

66. King SL, Lebert J, Karpisek LA, Phillips A, Neal T, Kosyluk K. Characterizing User Experiences With an SMS Text Messaging-Based mHealth Intervention: Mixed Methods Study. JMIR Form Res. May 3, 2022;6(5):e35699. [doi: ] [Medline: 35503524]

68. Chan K yi, Yeung N yiu, Mo P han, Yang X. Common stressors, coping processes, and professional help-seeking of medical professionals in Hong Kong: A qualitative study. J Health Psychol. Jul 2024;29(8):891-904. [doi: ]

69. Cliffe B, Stokes Z, Stallard P. The Acceptability of a Smartphone App (BlueIce) for University Students Who Self-harm. Arch Suicide Res. 2023;27(2):565-581. [doi: ] [Medline: 34983335]

70. Dela Cruz ENM, Marcelo RCA, Naling BYM, Ty WEG. “Hello, can you hear me?”: Narratives of online mental health counselling among Filipino adults during the pandemic. Couns and Psychother Res. Mar 2023;23(1):164-175. [doi: ]

71. Eccles H, Nannarone M, Lashewicz B, et al. Barriers to the Use of Web-Based Mental Health Programs for Preventing Depression: Qualitative Study. JMIR Form Res. Jul 15, 2021;5(7):e16949. [doi: ] [Medline: 34264195]

72. Fisher A, Corrigan E, Cross S, et al. Decision-making about uptake and engagement with digital mental health services: a qualitative exploration of service user perspectives. Clin Psychol (Aust Psychol Soc). Jan 2, 2024;28(1):37-48. [doi: ]

73. Hoffman BD, Oppert ML, Owen M. Understanding young adults’ attitudes towards using AI chatbots for psychotherapy: The role of self-stigma. Computers in Human Behavior: Artificial Humans. Aug 2024;2(2):100086. [doi: ]

74. Jackson HM, Gulliver A, Hasking P, et al. Exploring student preferences for implementing a digital mental health intervention in a university setting: Qualitative study within a randomised controlled trial. Digit HEALTH. 2024;10:20552076241277175. [doi: ] [Medline: 39224795]

75. Jardine J, Nadal C, Robinson S, Enrique A, Hanratty M, Doherty G. Between Rhetoric and Reality: Real-world Barriers to Uptake and Early Engagement in Digital Mental Health Interventions. ACM Trans Comput-Hum Interact. Apr 30, 2024;31(2):1-59. [doi: ]

76. Karwig G, Chambers D. E-mental health on-campus: college students’ views of online help-seeking. 2016.

77. Kim H, Kim M, Heo J in, Lee S, Kim H, Jung D. Understanding university students’ experiences on multi-domain help seeking platform “fruto”: vignettes study. Presented at: CHI EA ’25: Proceedings of the Extended Abstracts of the CHI Conference on Human Factors in Computing Systems; Apr 26 to May 1, 2025:1-8; Yokohama Japan. [doi: ]

78. Levin ME, Stocke K, Pierce B, Levin C. Do College Students Use Online Self-Help? A Survey of Intentions and Use of Mental Health Resources. J College Stud Psychother. Jul 3, 2018;32(3):181-198. [doi: ]

79. Mamdouh M, Tai AMY, Westenberg JN, et al. Egyptian Students Open to Digital Mental Health Care: Cross-Sectional Survey. JMIR Form Res. Mar 21, 2022;6(3):e31727. [doi: ] [Medline: 35311692]

80. McCall T, Foster M, Schwartz TA. Attitudes Toward Seeking Mental Health Services and Mobile Technology to Support the Management of Depression Among Black American Women: Cross-Sectional Survey Study. J Med Internet Res. Jul 19, 2023;25:e45766. [doi: ] [Medline: 37467027]

81. McCarthy K, Horwitz AG. Attitudes and barriers to mobile mental health interventions among first-year college students: a mixed-methods study. J Am Coll Health. Dec 2025;73(10):3931-3940. [doi: ] [Medline: 39868744]

82. Pretorius C, Chambers D, Cowan B, Coyle D. Young People Seeking Help Online for Mental Health: Cross-Sectional Survey Study. JMIR Ment Health. Aug 26, 2019;6(8):e13524. [doi: ] [Medline: 31452519]

83. Tickell A, Fonagy P, Hajdú K, Obradović S, Pilling S. “Am I really the priority here?”: help-seeking experiences of university students who self-harmed. BJPsych Open. Feb 1, 2024;10(2):e40. [doi: ] [Medline: 38297500]

84. Wallin EEK, Mattsson S, Olsson EMG. The Preference for Internet-Based Psychological Interventions by Individuals Without Past or Current Use of Mental Health Treatment Delivered Online: A Survey Study With Mixed-Methods Analysis. JMIR Ment Health. Jun 14, 2016;3(2):e25. [doi: ] [Medline: 27302200]

85. Wallin E, Maathz P, Parling T, Hursti T. Self-stigma and the intention to seek psychological help online compared to face-to-face. J Clin Psychol. Jul 2018;74(7):1207-1218. [doi: ] [Medline: 29315545]

*Risk of bias assessment of the included studies (first half)*

| Criteria/Score | S01 | S02 | S03 | S04 | S05 | S06 | S07 | S08 | S09 | S10 | S11 |
| --- | --- | --- | --- | --- | --- | --- | --- | --- | --- | --- | --- |
| Theoretical or conceptual underpinning to the research | 1 | 3 | 2 | 3 | 3 | 2 | 2 | 3 | 1 | 2 | 2 |
| Statement of research aim/s | 3 | 3 | 3 | 3 | 3 | 3 | 3 | 3 | 3 | 3 | 3 |
| Clear description of research setting and target population | 2 | 3 | 2 | 2 | 2 | 3 | 3 | 2 | 3 | 2 | 2 |
| The study design is appropriate to address the stated research aim/s | 3 | 3 | 3 | 3 | 3 | 3 | 3 | 3 | 3 | 3 | 3 |
| Appropriate sampling to address the research aim/s | 2 | 3 | 2 | 3 | 3 | 3 | 3 | 3 | 3 | 3 | 3 |
| Rationale for choice of data collection tool/s | 3 | 3 | 3 | 3 | 3 | 1 | 2 | 3 | 0 | 3 | 3 |
| The format and content of data collection tool is appropriate to address the stated research aim/s | 3 | 2 | 3 | 3 | 3 | 2 | 3 | 3 | 1 | 2 | 2 |
| Description of data collection procedure | 3 | 2 | 3 | 3 | 3 | 1 | 3 | 2 | 1 | 2 | 3 |
| Recruitment data provided | 3 | 3 | 2 | 3 | 2 | 2 | 1 | 2 | 2 | 2 | 2 |
| Justification for analytic method selected | 1 | 1 | 3 | 2 | 1 | 2 | 1 | 1 | 1 | 3 | 0 |
| The method of analysis was appropriate to answer the research aim/s | 2 | 2 | 2 | 2 | 2 | 3 | 3 | 3 | 2 | 3 | 3 |
| Evidence that the research stakeholders have been considered in research design or conduct | 0 | 0 | 2 | 0 | 0 | 1 | 1 | 2 | 0 | 2 | 1 |
| Strengths and limitations critically discussed | 2 | 2 | 3 | 2 | 3 | 3 | 3 | 2 | 1 | 1 | 2 |
| **Total** | 28 | 30 | 33 | 32 | 31 | 29 | 31 | 32 | 21 | 31 | 29 |
| **Score as Percentage (%)** | 72 | 77 | 85 | 82 | 79 | 74 | 79 | 82 | 54 | 79 | 74 |

*Risk of bias assessment of the included studies (second half)*

| Criteria/Score | S12 | S13 | S14 | S15 | S16 | S17 | S18 | S19 | S20 | S21 | S22 |
| --- | --- | --- | --- | --- | --- | --- | --- | --- | --- | --- | --- |
| Theoretical or conceptual underpinning to the research | 1 | 2 | 2 | 2 | 2 | 2 | 2 | 2 | 1 | 2 | 2 |
| Statement of research aim/s | 3 | 3 | 3 | 3 | 3 | 3 | 3 | 3 | 3 | 3 | 3 |
| Clear description of research setting and target population | 3 | 3 | 2 | 2 | 3 | 2 | 2 | 3 | 2 | 3 | 3 |
| The study design is appropriate to address the stated research aim/s | 3 | 3 | 3 | 3 | 3 | 2 | 3 | 3 | 3 | 3 | 3 |
| Appropriate sampling to address the research aim/s | 3 | 3 | 3 | 3 | 3 | 3 | 3 | 3 | 2 | 3 | 3 |
| Rationale for choice of data collection tool/s | 3 | 1 | 1 | 3 | 3 | 2 | 3 | 3 | 2 | 2 | 2 |
| The format and content of data collection tool is appropriate to address the stated research aim/s | 3 | 2 | 3 | 3 | 2 | 3 | 2 | 3 | 2 | 3 | 1 |
| Description of data collection procedure | 3 | 3 | 1 | 3 | 3 | 3 | 2 | 2 | 3 | 3 | 1 |
| Recruitment data provided | 1 | 3 | 3 | 3 | 2 | 3 | 3 | 3 | 1 | 1 | 1 |
| Justification for analytic method selected | 1 | 1 | 0 | 2 | 2 | 2 | 0 | 3 | 3 | 3 | 2 |
| The method of analysis was appropriate to answer the research aim/s | 2 | 2 | 2 | 2 | 2 | 1 | 2 | 2 | 2 | 2 | 2 |
| Evidence that the research stakeholders have been considered in research design or conduct | 1 | 0 | 2 | 2 | 1 | 2 | 1 | 1 | 0 | 0 | 0 |
| Strengths and limitations critically discussed | 2 | 3 | 1 | 3 | 2 | 3 | 2 | 3 | 3 | 3 | 2 |
| **Total** | 29 | 29 | 26 | 34 | 31 | 31 | 28 | 34 | 27 | 31 | 25 |
| **Score as Percentage (%)** | 74 | 74 | 67 | 87 | 79 | 79 | 72 | 87 | 69 | 79 | 64 |
