# Supplementary material for: Factors Influencing the Initiation and Continued Engagement of Digital Mental Health Tools Among Adults: Theory of Planned Behavior–Informed Systematic Review
Source: JMIR Ment Health. 2026 May 15;13:e88731. doi: 10.2196/88731 (PMC13179054; doi:10.2196/88731)
Supplement: Multimedia Appendix 4 [file mental-v13-e88731-s004.docx]

**Appendix 4**

*General Descriptive Features of 22 Studies*

| Study IDs | First Author (Year) | Country | Age Group | Gender Identity | Ethnic/Cultural Group | Sample Size | Study Design | DMHT type | Target MH Concern |
| --- | --- | --- | --- | --- | --- | --- | --- | --- | --- |
| S01 | Chan (2024) [68] | Hong Kong | 28 to 52 (mean = 34.8) | Male, Female and Non-Binary | NA | 10 | Semi-structured interviews | NA | General Wellbeing & Distress |
| S02 | Cliffe (2023) [69] | UK | 18 to 31 (mean 20.6) | Male, Female and Non-Binary | White, Asian/Asian British, Mixed | 25 | Semi-structured interviews | Smartphone APP (BlueIce) | Self-Harm |
| S03 | Dela Cruz (2023) [70] | Philippines | 20 to 32 | Male, Female and Non-Binary | Filipino | 11 | Semi-structured interviews | Online mental health counselling | General Wellbeing |
| S04 | Eccles (2021) [71] | Canada | 20 to 67 (mean 40.6) | Male | Participant from different location in Canada | 70 | Semi-structured interviews | Web-based mental health programs | Depression |
| S05 | Fisher (2024) [72] | Australia | 27 to 74 (mean 52.75) | Male, Female and Non-Binary | Participant from different location in Australia | 20 | Semi-structured interviews | MindSpot, digital and telehealth mental health service | Anxiety & Depression |
| S06 | Hoffman (2024) [73] | Australia | 18 to 24 (mean 21.46) | Male, Female and Non-Binary | Australian ethnicity, Italian, Greek, Indian, and Other | 109 | Survey | AI chatbot psychotherapy | General Wellbeing |
| S07 | Jackson (2024) [74] | Australia | Mean age 20.63 | Male, Female and Non-Binary | Most participants were domestic students (83.5%) | 115 | Semi-structured interviews and post intervention surveys | Video based mental health intervention | General Wellbeing |
| S08 | Jardine (2024) [75] | US and UK | 18 to 64 | Male, Female and Non-Binary | White, Asian, Black, Mixed, Indian, Latino/Hispanic, Native American/Alaskan native, and south pacific islander | 205 | Semi-structured online interviews | SliverCloud by Amwell, a digital mental health platform | Anxiety, Depression & General Wellbeing |
| S09 | Karwig (2016) [76] | Ireland | 18 to 22 | Male, Female and Non-Binary | The majority of respondents were Irish (85%) | 5556 | Online survey and group interviews | NA | General Wellbeing |
| S10 | Kim (2025) [77] | Korea | 19 to 31 | Male, Female and Non-Binary | NA | 16 | Semi-structured interviews | Mobile App | General Wellbeing |
| S11 | King (2022) [66] | US | 18 to 59 | Male, Female and Non-Binary | White, Middle Eastern or Arab American, Asian American or Asian, Black or African American, Pacific Islander. | 14 | Qualitative interviews and user survey | SMS text messaging based mHealth intervention | General Wellbeing |
| S12 | Kodish (2023) [28] | US | Average respondent age 45.2 | Male, Female and Non-Binary | White, Asian American, Latinx, Multiracial, Middle Eastern/North African and Black/African American. | 35 | A modified, three-round Delphi Survey | NA | General Wellbeing |
| S13 | Levin (2018) [78] | US | 18 to 45, Average = 20.08 | Male, Female and Non-Binary | White, Hispanic or Latino, Asian, Black or African American, American Indian/ Alaska Native, Native Hawaiian or other Pacific Islander, Multiracial. | 389 | Survey | Mobile App | General Wellbeing |
| S14 | Mamdouh (2022) [79] | Egypt | 18 to 25 (Mean=20.5) | Male, Female and Non-Binary | NA | 778 | Survey | General Electronic mental health (EMH) | General Wellbeing |
| S15 | McCall (2023) [80] | US | 18 to 107 (mean = 44.8) | Female | All identify as either Black or African American or multiracial. | 395 | Survey | Mobile Technology (i.e., Voice call, video call, SMS text messaging, and mobile app) | Depression |
| S16 | McCarthy (2025) [81] | US | 18 to 25 | Male, Female and Non-Binary | non-Hispanic white, | 351 | Semi-structured interviews | Mobile mental health applications | Anxiety & Depression |
| S17 | Mwaka (2025) [61] | Uganda | 21 to 25 (median age = 24) | Male, Female and Non-Binary | NA | 50 | Face to face focus group discussions | NA | General Wellbeing |
| S18 | Pretorius (2019) [82] | Ireland | !8 to 25 (mean age = 20.68) | Male, Female and Non-Binary | Participant from different location in Ireland | 1308 | Survey | Youth mental health-related website (ReachOut Ireland, SpunOut, and BodyWhys) | General Wellbeing |
| S19 | Tan (2025) [62] | Singapore | 21 to 63 years (mean age = 33.1) | Male, Female and Non-Binary | Chinese (83%), Malay (9%), Indian (4%), other (4%) | 23 | Semi-structured interviews | Mobile App | General Wellbeing |
| S20 | Tickell (2024) [83] | UK | NA | Male, Female and Non-Binary | White British or European (58%), Asian and Asian British (17%), Mixed ethnicity (8%), Black British (8%), South American (8%) | 12 | Semi-structured interviews | NA | General Wellbeing |
| S21 | Wallin (2016) [84] | Sweden | Mean age 52.3 | Male, Female and Non-Binary | Born in Sweden (91.3%), Born outside of Sweden (8.7%) | 439 | Survey | Internet-based psychological interventions | General Wellbeing |
| S22 | Wallin (2018) [85] | Sweden | Mean age 34.9 | Male, Female and Non-Binary | Born in Sweden (83.35%), Born outside of Sweden (16.65%) | 462 | Survey | Internet-based psychological interventions | General Wellbeing |

References:

28. Kodish T, Schueller SM, Lau AS. Barriers and strategies to improve digital mental health intervention uptake among college students of color: A modified Delphi study. Journal of Behavioral and Cognitive Therapy. Mar 2023;33(1):10-23. [doi: ]

61. Mwaka ES, Bazzeketa D, Mirembe J, Emoru RD, Twimukye A, Kivumbi A. Barriers to and enhancement of the utilization of digital mental health interventions in low-resource settings: Perceptions of young people in Uganda. Digit HEALTH. 2025;11:20552076251321698. [doi: ] [Medline: 39963503]

62. Tan CJY, Müller AM, Rajendram P, Subramaniam M. Factors Affecting the Adoption of Mental Health Apps in Workplaces: A Qualitative Study. J technol behav sci. 2025. [doi: 10.1007/s41347-025-00524-z]

66. King SL, Lebert J, Karpisek LA, Phillips A, Neal T, Kosyluk K. Characterizing User Experiences With an SMS Text Messaging-Based mHealth Intervention: Mixed Methods Study. JMIR Form Res. May 3, 2022;6(5):e35699. [doi: ] [Medline: 35503524]

68. Chan K yi, Yeung N yiu, Mo P han, Yang X. Common stressors, coping processes, and professional help-seeking of medical professionals in Hong Kong: A qualitative study. J Health Psychol. Jul 2024;29(8):891-904. [doi: ]

69. Cliffe B, Stokes Z, Stallard P. The Acceptability of a Smartphone App (BlueIce) for University Students Who Self-harm. Arch Suicide Res. 2023;27(2):565-581. [doi: ] [Medline: 34983335]

70. Dela Cruz ENM, Marcelo RCA, Naling BYM, Ty WEG. “Hello, can you hear me?”: Narratives of online mental health counselling among Filipino adults during the pandemic. Couns and Psychother Res. Mar 2023;23(1):164-175. [doi: ]

71. Eccles H, Nannarone M, Lashewicz B, et al. Barriers to the Use of Web-Based Mental Health Programs for Preventing Depression: Qualitative Study. JMIR Form Res. Jul 15, 2021;5(7):e16949. [doi: ] [Medline: 34264195]

72. Fisher A, Corrigan E, Cross S, et al. Decision-making about uptake and engagement with digital mental health services: a qualitative exploration of service user perspectives. Clin Psychol (Aust Psychol Soc). Jan 2, 2024;28(1):37-48. [doi: ]

73. Hoffman BD, Oppert ML, Owen M. Understanding young adults’ attitudes towards using AI chatbots for psychotherapy: The role of self-stigma. Computers in Human Behavior: Artificial Humans. Aug 2024;2(2):100086. [doi: ]

74. Jackson HM, Gulliver A, Hasking P, et al. Exploring student preferences for implementing a digital mental health intervention in a university setting: Qualitative study within a randomised controlled trial. Digit HEALTH. 2024;10:20552076241277175. [doi: ] [Medline: 39224795]

75. Jardine J, Nadal C, Robinson S, Enrique A, Hanratty M, Doherty G. Between Rhetoric and Reality: Real-world Barriers to Uptake and Early Engagement in Digital Mental Health Interventions. ACM Trans Comput-Hum Interact. Apr 30, 2024;31(2):1-59. [doi: ]

76. Karwig G, Chambers D. E-mental health on-campus: college students’ views of online help-seeking. 2016.

77. Kim H, Kim M, Heo J in, Lee S, Kim H, Jung D. Understanding university students’ experiences on multi-domain help seeking platform “fruto”: vignettes study. Presented at: CHI EA ’25: Proceedings of the Extended Abstracts of the CHI Conference on Human Factors in Computing Systems; Apr 26 to May 1, 2025:1-8; Yokohama Japan. [doi: ]

78. Levin ME, Stocke K, Pierce B, Levin C. Do College Students Use Online Self-Help? A Survey of Intentions and Use of Mental Health Resources. J College Stud Psychother. Jul 3, 2018;32(3):181-198. [doi: ]

79. Mamdouh M, Tai AMY, Westenberg JN, et al. Egyptian Students Open to Digital Mental Health Care: Cross-Sectional Survey. JMIR Form Res. Mar 21, 2022;6(3):e31727. [doi: ] [Medline: 35311692]

80. McCall T, Foster M, Schwartz TA. Attitudes Toward Seeking Mental Health Services and Mobile Technology to Support the Management of Depression Among Black American Women: Cross-Sectional Survey Study. J Med Internet Res. Jul 19, 2023;25:e45766. [doi: ] [Medline: 37467027]

81. McCarthy K, Horwitz AG. Attitudes and barriers to mobile mental health interventions among first-year college students: a mixed-methods study. J Am Coll Health. Dec 2025;73(10):3931-3940. [doi: ] [Medline: 39868744]

82. Pretorius C, Chambers D, Cowan B, Coyle D. Young People Seeking Help Online for Mental Health: Cross-Sectional Survey Study. JMIR Ment Health. Aug 26, 2019;6(8):e13524. [doi: ] [Medline: 31452519]

83. Tickell A, Fonagy P, Hajdú K, Obradović S, Pilling S. “Am I really the priority here?”: help-seeking experiences of university students who self-harmed. BJPsych Open. Feb 1, 2024;10(2):e40. [doi: ] [Medline: 38297500]

84. Wallin EEK, Mattsson S, Olsson EMG. The Preference for Internet-Based Psychological Interventions by Individuals Without Past or Current Use of Mental Health Treatment Delivered Online: A Survey Study With Mixed-Methods Analysis. JMIR Ment Health. Jun 14, 2016;3(2):e25. [doi: ] [Medline: 27302200]

85. Wallin E, Maathz P, Parling T, Hursti T. Self-stigma and the intention to seek psychological help online compared to face-to-face. J Clin Psychol. Jul 2018;74(7):1207-1218. [doi: ] [Medline: 29315545]
